# Supplementary figures and images for: Trait Variation in Yeast Is Defined by Population History
Source: PLoS Genet. 2011 Jun 16;7(6):e1002111. doi: 10.1371/journal.pgen.1002111 (PMC3116910; doi:10.1371/journal.pgen.1002111)

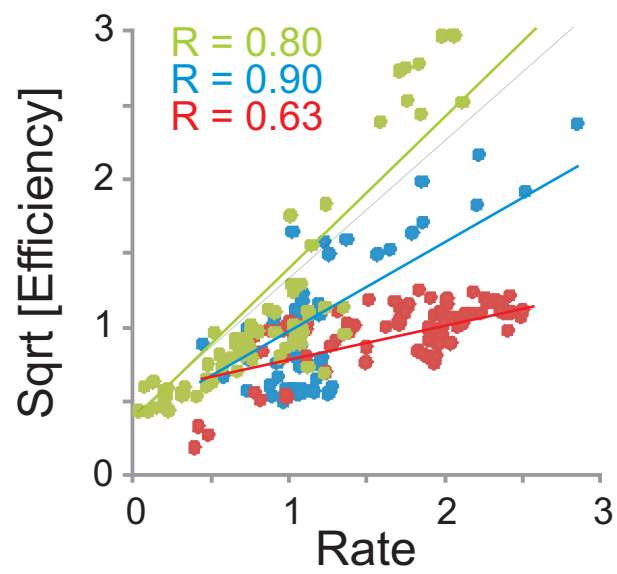

Figure S1 Warringer *et al*

Supplement: Figure S1 — The correlation between proliferative rate and efficiency is environment dependent. The linear correlation (Pearson correlation coefficients, r) between the proliferative rate and the square root of the proliferative efficiency was plotted for each environment separately. Three sample environments are displayed. Red = clotrimazole (3 µM) exposure, blue = maltose (8%) growth, green = 1,2,4-aminotriazole (300 mM) exposure. No evidence of a general adherence to a 1∶1 correlation was observed, which would be expected if the rate was maximized in each environment and restricted only by biomass yield. (PDF) [file pgen.1002111.s002.pdf]

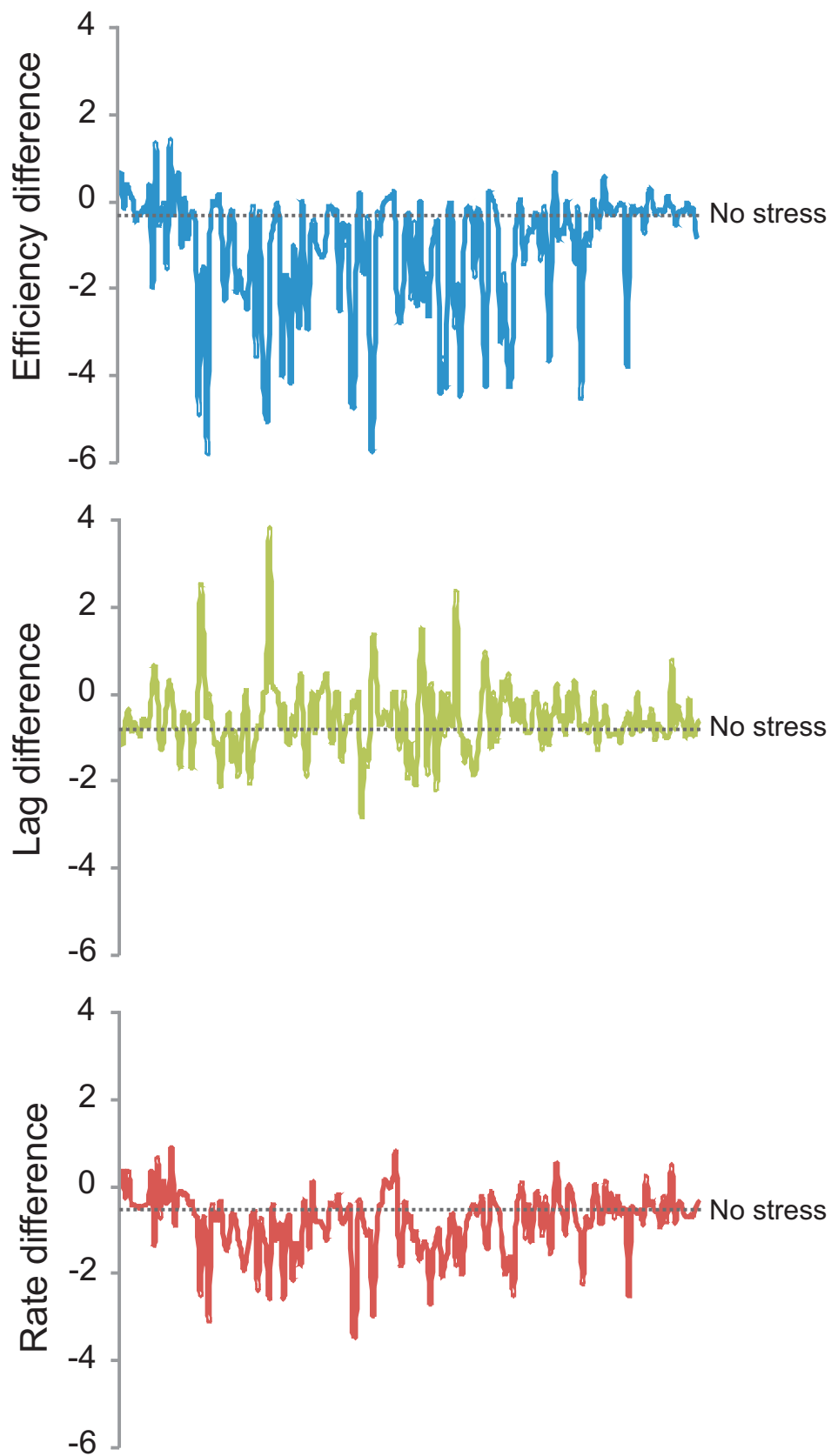

Figure S2 Warringer *et al*

Supplement: Figure S2 — The S. cerevisiae, S. mikatae and S. paradoxus clade shows superior performance to the S. kudriavzevii, S. bayanus and S. arboricolus clade in a wide range of environments. Trait averages within the S. cerevisiae/S. mikatae/S. paradoxus clade and the S. kudriavzevii/S. bayanus/S. arboricolus clade were calculated for each trait separately, and the clade difference was calculated as (S. kudriavzevii/S. bayanus/S. arboricolus) - (S. cerevisiae/S. mikatae/S. paradoxus). Lag, rate and efficiency traits are displayed separately. Dotted lines indicated with “No stress” correspond to differences in basal conditions. (PDF) [file pgen.1002111.s003.pdf]

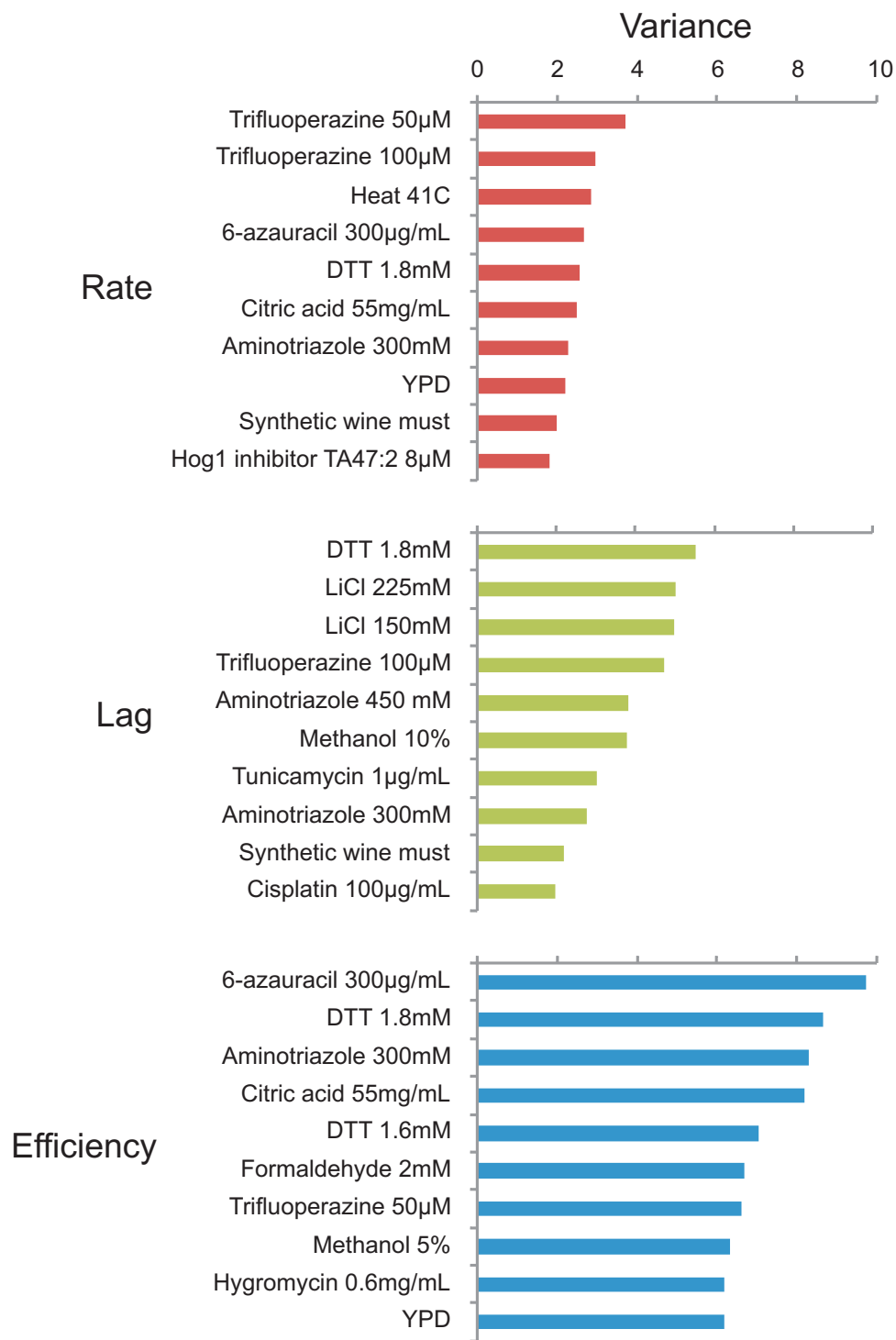

Figure S3 Warringer *et al*

Supplement: Figure S3 — Traits varying between Saccharomyces sensu stricto species. The trait variance between Saccharomyces sensu stricto species was calculated for each trait separately using species trait averages. The top ten environments with highest between-species trait variance for each proliferative measure are displayed. (PDF) [file pgen.1002111.s004.pdf]

A

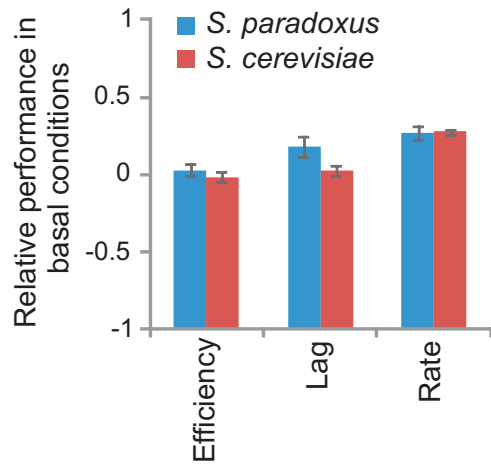

C

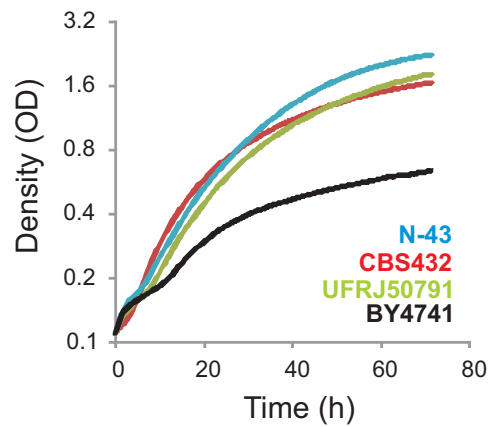

D

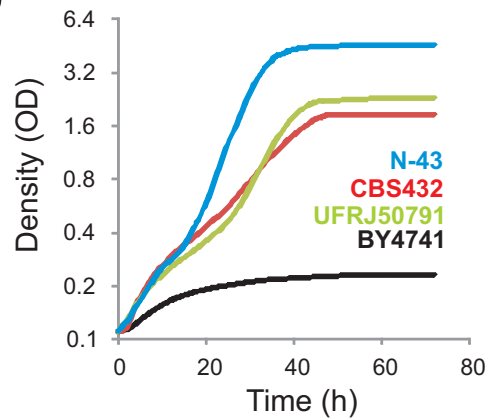

B

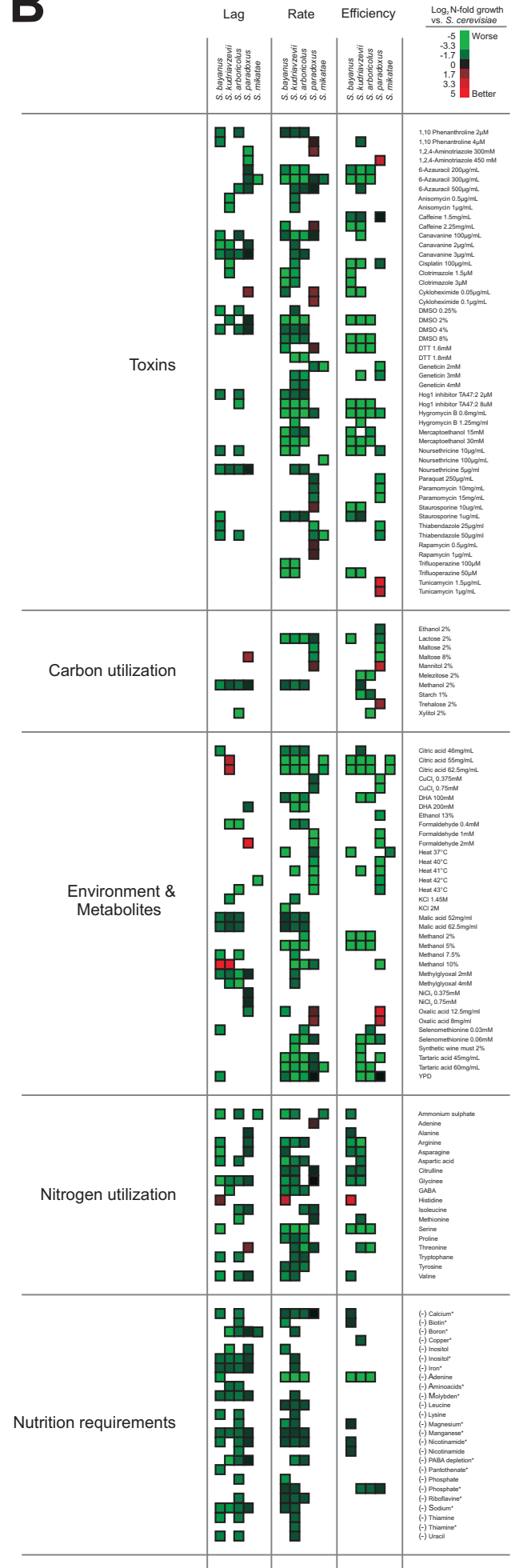Figure S4 Warringer *et al*

Supplement: Figure S4 — S. cerevisiae shows superior performance to other Saccharomyces sensu stricto species in a wide range of environments. A) Average proliferative ability in basal conditions in S. paradoxus and S. cerevisiae in relation to the S. cerevisiae reference strain (BY4741, Log2 scale). Error bars correspond to standard errors. No significant (Bonferroni corrected Student's t-test, p>0.25) differences were observed. B) The difference in proliferation between each of S. arboricolus (n = 3), S. bayanus (n = 3), S. kudriavzevii (n = 4), S. mikatae (n = 2) and S. paradoxus (n = 35) and S. cerevisiae (n = 35) was determined separately for each proliferative measure. Significant differences at p<0.1 (Student's t-test, Bonferroni correction) are displayed. Green indicates proliferation inferior to that of S. cerevisiae, red indicates proliferation superior to that of S. cerevisiae. * pre-culture performed using the same nitrogen source as in the experiment (see Text S1) C) Proliferation of the S. paradoxus Far East (N-43), European (CBS432) and American (UFRJ50791) populations as compared to that of S. cerevisiae (BY4741), using mannitol as carbon source. D) Proliferation of the S. paradoxus Far East (N-43), European (N-17) and American (A12) populations as compared to that of S. cerevisiae (BY4741) during oxalic acid (12.5 mg/mL) exposure. (PDF) [file pgen.1002111.s005.pdf]

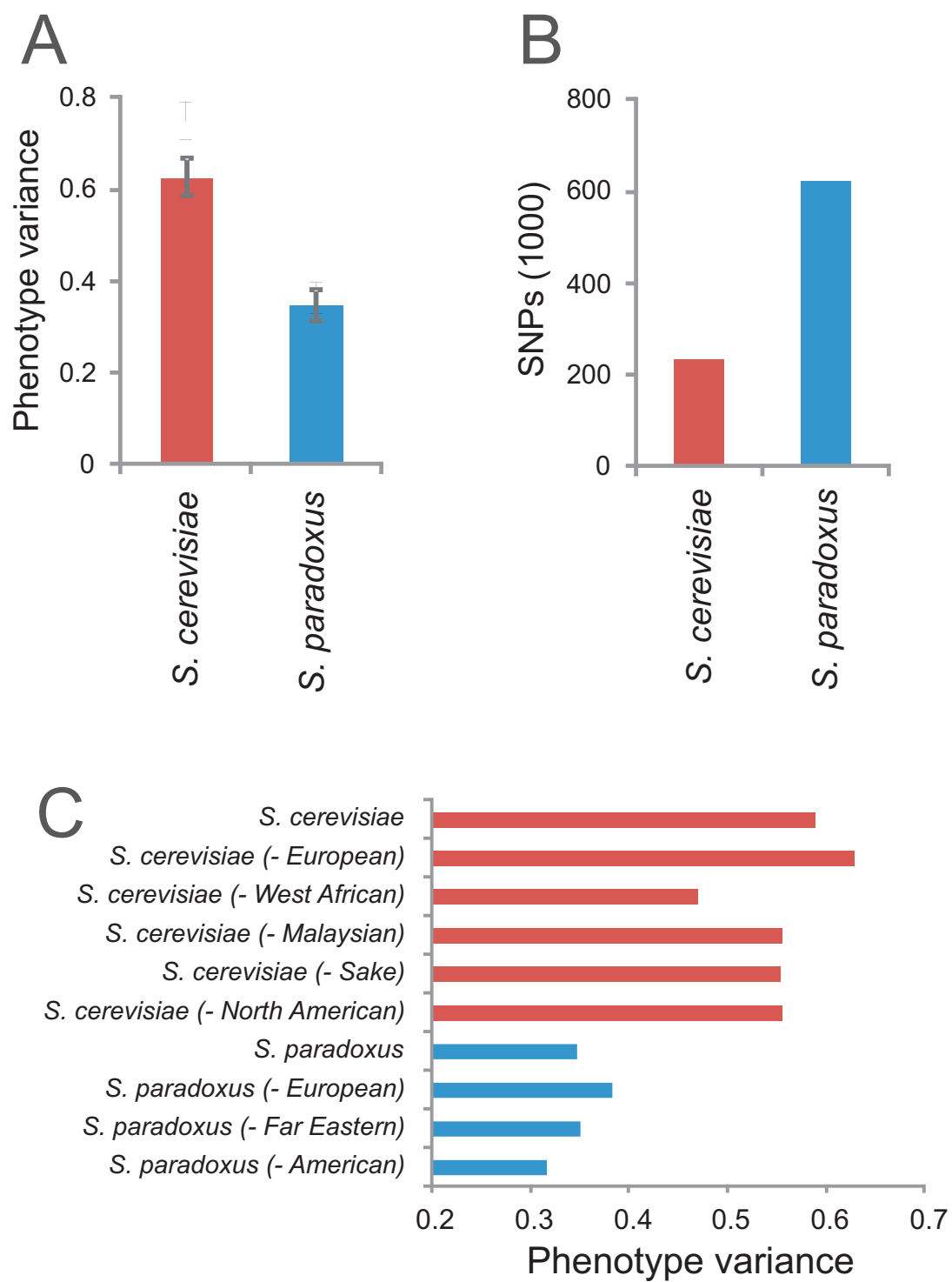

Figure S5 Warringer *et al*

Supplement: Figure S5 — Trait variation in S. cerevisiae exceeds trait variation in S. paradoxus. A) Trait variance within S. cerevisiae and within S. paradoxus was calculated for each trait separately and a mean over all traits was formed. Error bars represent standard errors. B) Genetic variation within S. cerevisiae and within S. paradoxus [16], represented by the total number of SNPs within each species. C) To determine whether the difference in within-species trait variation between S. cerevisiae and S. paradoxus is due to population sub-structure effects, trait variance calculations, systematically and repeatedly excluding one individual population at a time, were performed. The difference between species was clear regardless of population excluded. (PDF) [file pgen.1002111.s006.pdf]

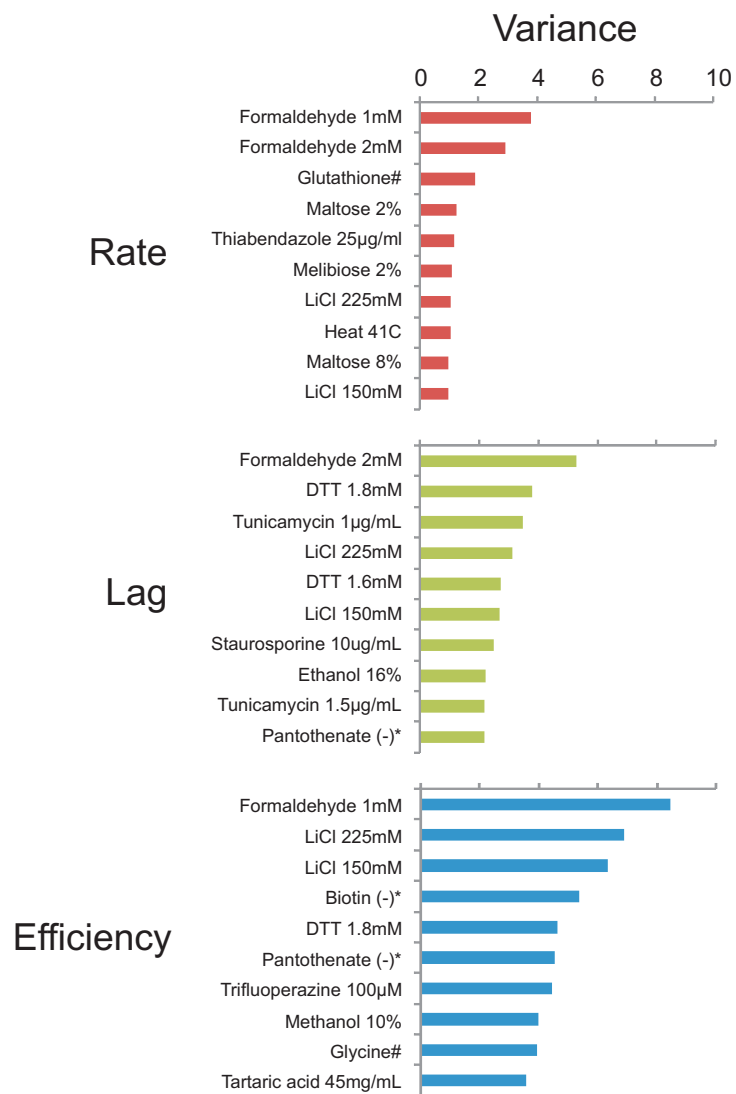

Figure S6 Warringer *et al*

Supplement: Figure S6 — Traits varying within S. cerevisiae. The trait variance over all S. cerevisiae isolates was calculated for each trait separately. Environments were ranked separately for each proliferative measure according to degree of variance. Top ten environments are displayed. (PDF) [file pgen.1002111.s007.pdf]

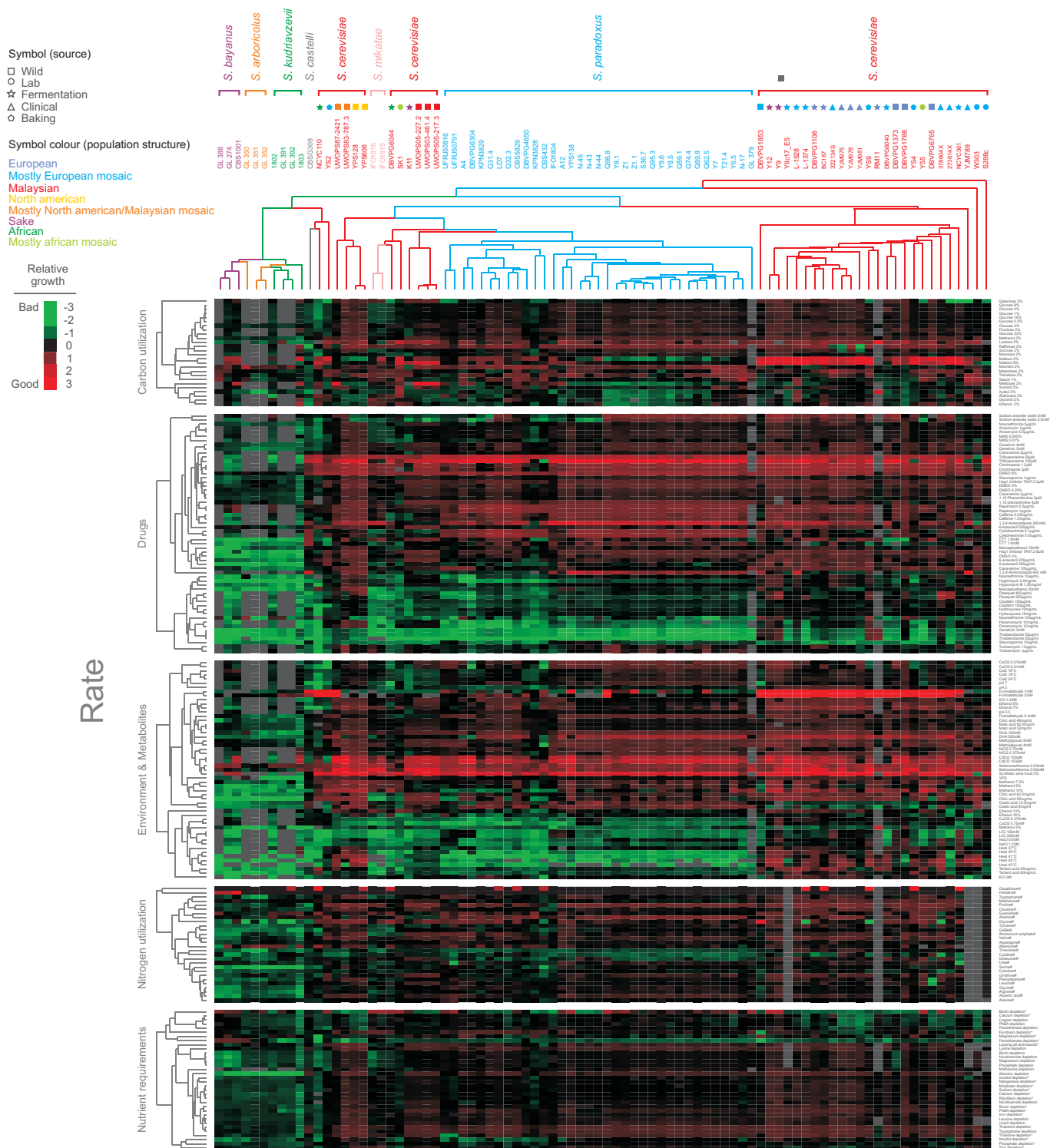

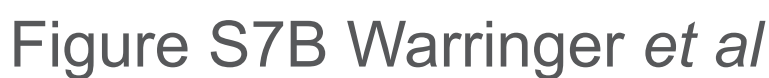Figure S7B Warringer *et al*

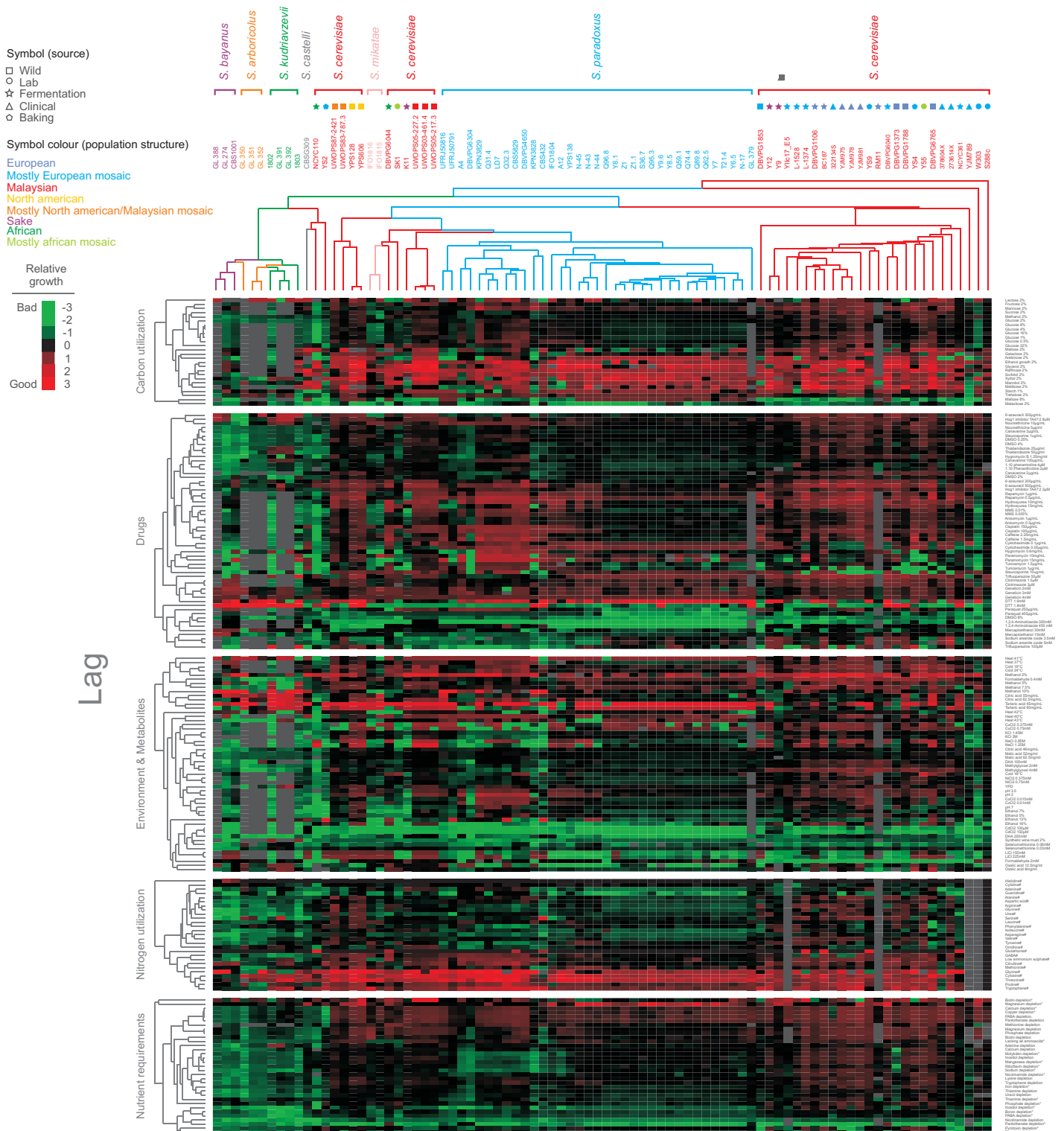

Figure S7C Warringer *et al*

Supplement: Figure S7 — Clustering of trait profiles of Saccharomyces sensu stricto isolates. Hierarchical clustering of all traits was performed using a centered Pearson correlation metric and average linkage mapping as described in the Materials and Methods. Species are indicated by line color. The heat map reflects the proliferation of each isolate in relation to either BY4741 or S288C. Green = inferior proliferation, red = superior proliferation, black = BY4741/S288C performance, grey = data not available. For S. cerevisiae, source habitats (symbols) and population structure [1] are indicated (symbol color). A) Proliferative rate traits B) Proliferative efficiency traits C) Proliferative lag traits. (PDF) [file pgen.1002111.s008.pdf]

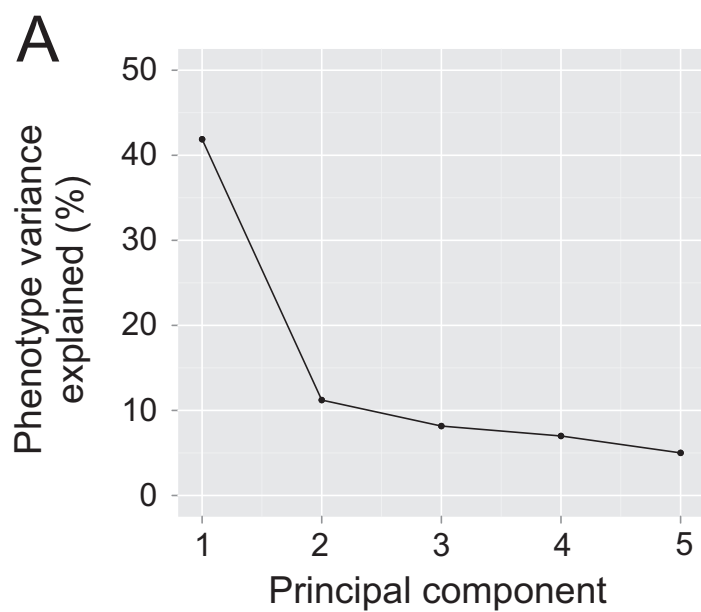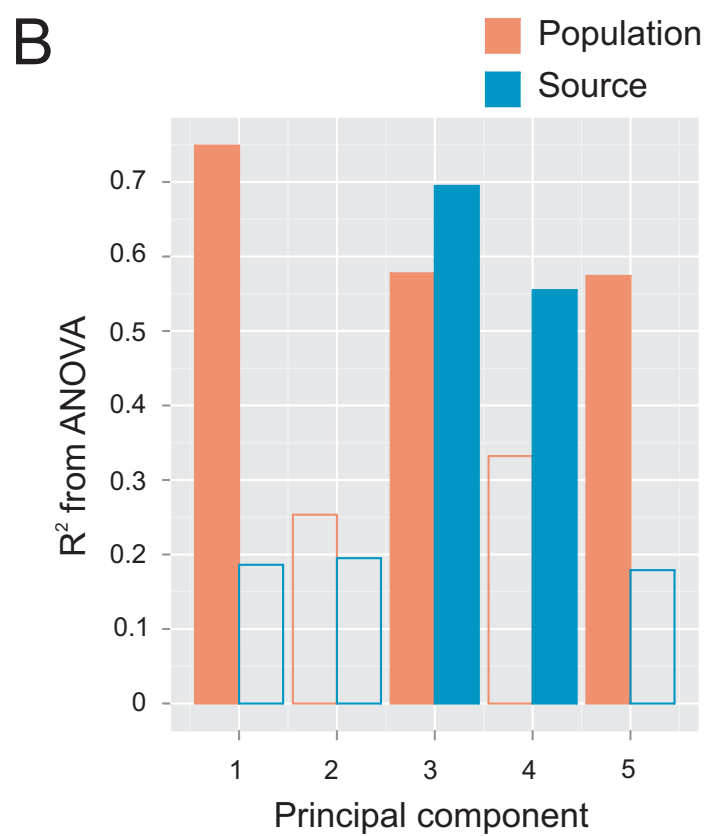

Figure S8 Warringer *et al*

Supplement: Figure S8 — Trait variation in S. cerevisiae is defined by population structure rather than source. To stringently evaluate the effect of population and source on trait variation within Saccharomyces cerevisiae, principal component analysis (PCA) followed by ANOVA was performed. Traits with one or more missing values were removed leaving 523 traits, and 21 clean lineage isolates, the latter independently categorized into five population and five source categories. Before PCA, strains were centered and scaled to unit variance. A) Scree plot of phenotypic variance (%) explained by each principal component. The plot shows that the phenotypic variation is high dimensional, with only the first principal component having substantial explanatory power on its own (42%). B) ANOVA was performed on the five first principal components individually, using population and source as regressors. The plot shows the fraction of variance in each principal component that is explained by population and source respectively. Filled bars = significant (p<0.01) effect of population/source, empty bars = non-significant effect of population/source. The principal component analysis shows that the first and overwhelmingly dominant principal component, explaining 42% of the trait variation, was very strongly influenced by population (ANOVA, r = 0.87, p = 1.1E-4) with no effect of source, whereas the following four components, together accounting for 31% of the variation, showed minor and comparable effects of population and source. Hence, the overall effect of population was substantially larger than the overall effect of source. (PDF) [file pgen.1002111.s009.pdf]

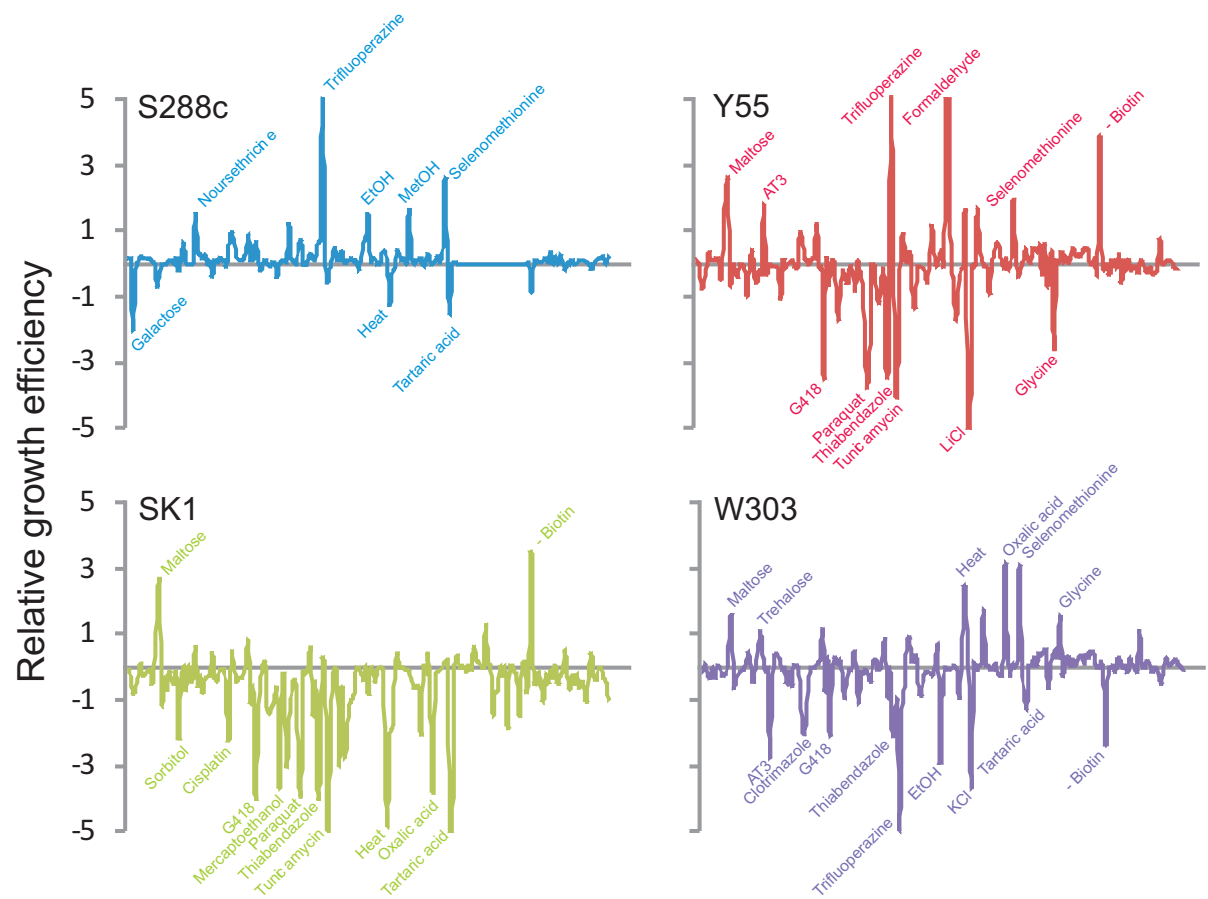

Figure S9 Warringer *et al*

Supplement: Figure S9 — S. cerevisiae lab strains have diverging trait profiles. The proliferative efficiency of the four commonly used S. cerevisiae lab strains, S288C, W303, SK1 and Y55, in relation to that of the reference strain BY4741, Log2 (isolate/BY4741), in ∼200 environments. Environments where a strong difference of the respective lab strain to BY4741 was observed are indicated with names. (PDF) [file pgen.1002111.s010.pdf]

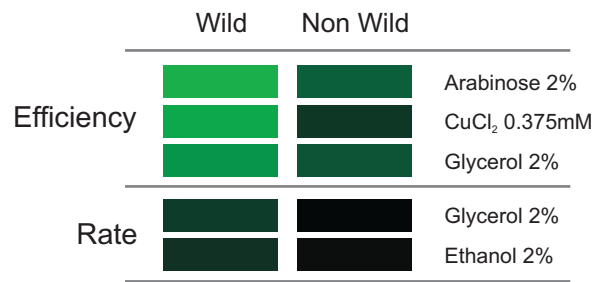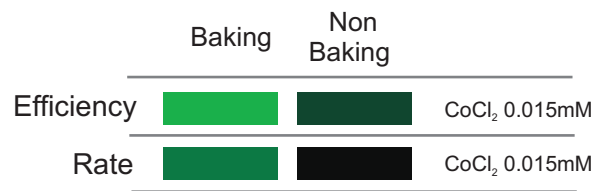

Figure S10 Warringer *et al*

Supplement: Figure S10 — Source dependent traits in S. cerevisiae. Traits that differ significantly (FDR = 2%) between isolates from one source classes and isolates from all other source classes. (PDF) [file pgen.1002111.s011.pdf]

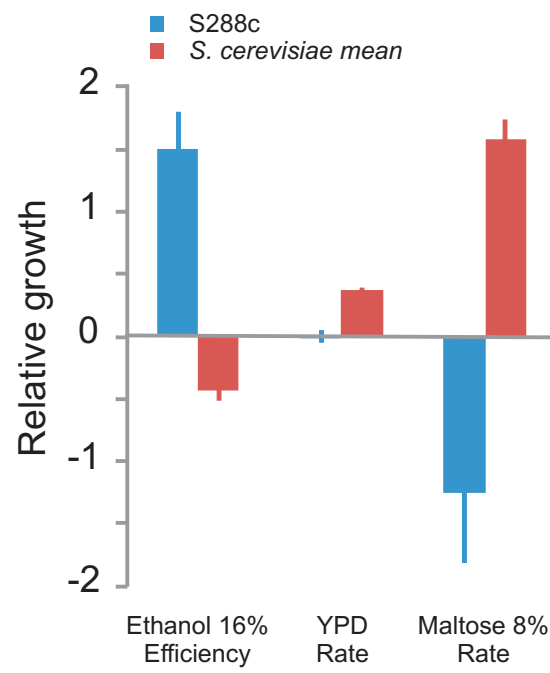

Figure S11 Warringer *et al*

Supplement: Figure S11 — Traits unique for the universal reference strain S288c. Traits for which S288C deviate significantly (p<0.1, Student's t-test, Bonferroni correction) from all other S. cerevisiae isolates. (PDF) [file pgen.1002111.s012.pdf]

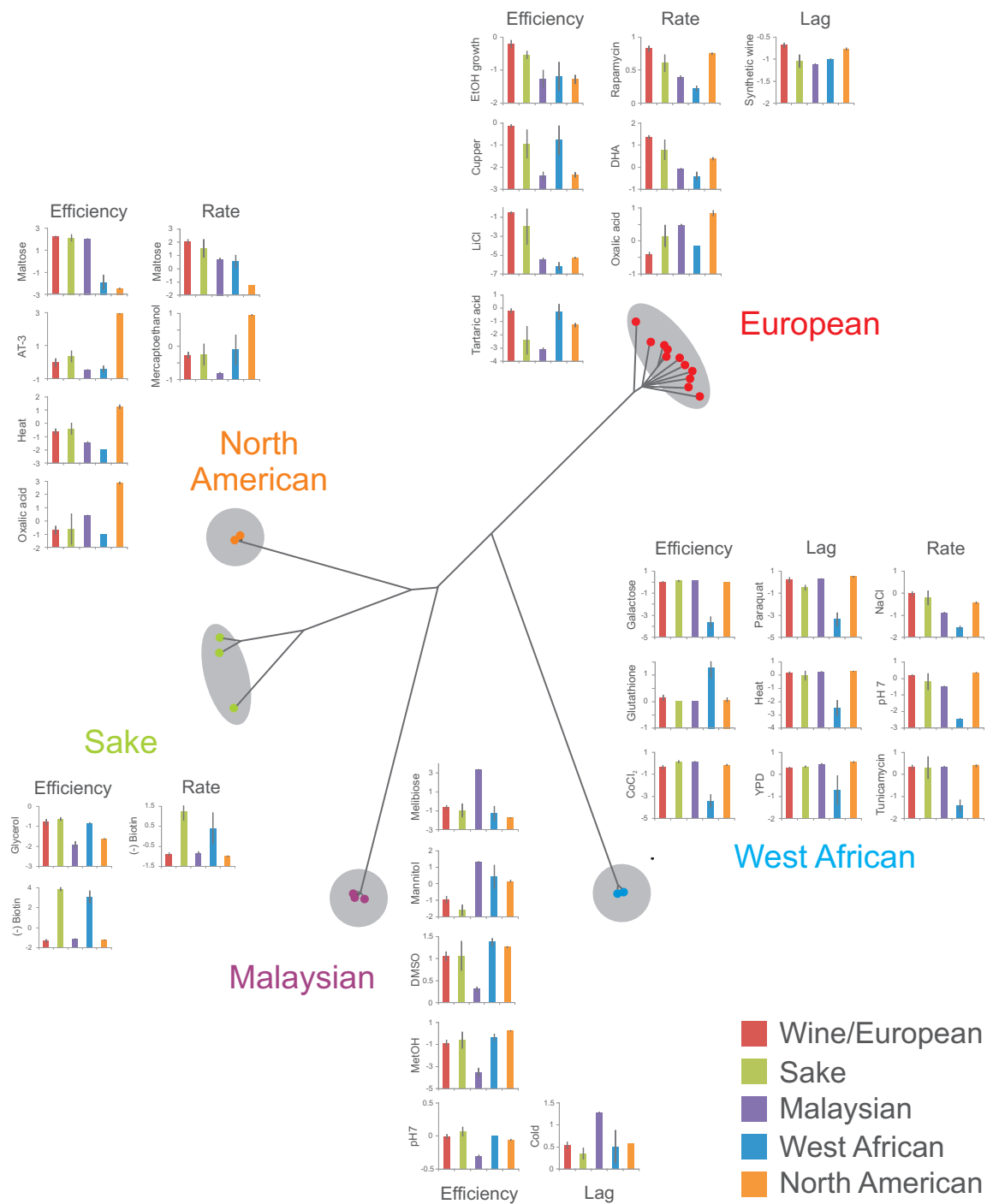

Figure S12 Warringer *et al*

Supplement: Figure S12 — Reconstructing the phenotypic history of S. cerevisiae. Population specific traits in S. cerevisiae were mapped onto a recently established population genomics tree based on low coverage genome sequence data [16]. Population specific traits were defined as environments where the performance of isolates in one population deviated significantly from isolates in other populations (FDR = 2%). Percentages indicate frequency of population specific traits in each population. Inset bar diagrams show a subset of population specific traits for each population. Bars represent trait averages with bar color indicating population and error bars representing standard errors. Total number of population specific phenotypes: West African = 190, European = 30, North American = 13, Malaysian = 13 and Sake = 3. (PDF) [file pgen.1002111.s013.pdf]

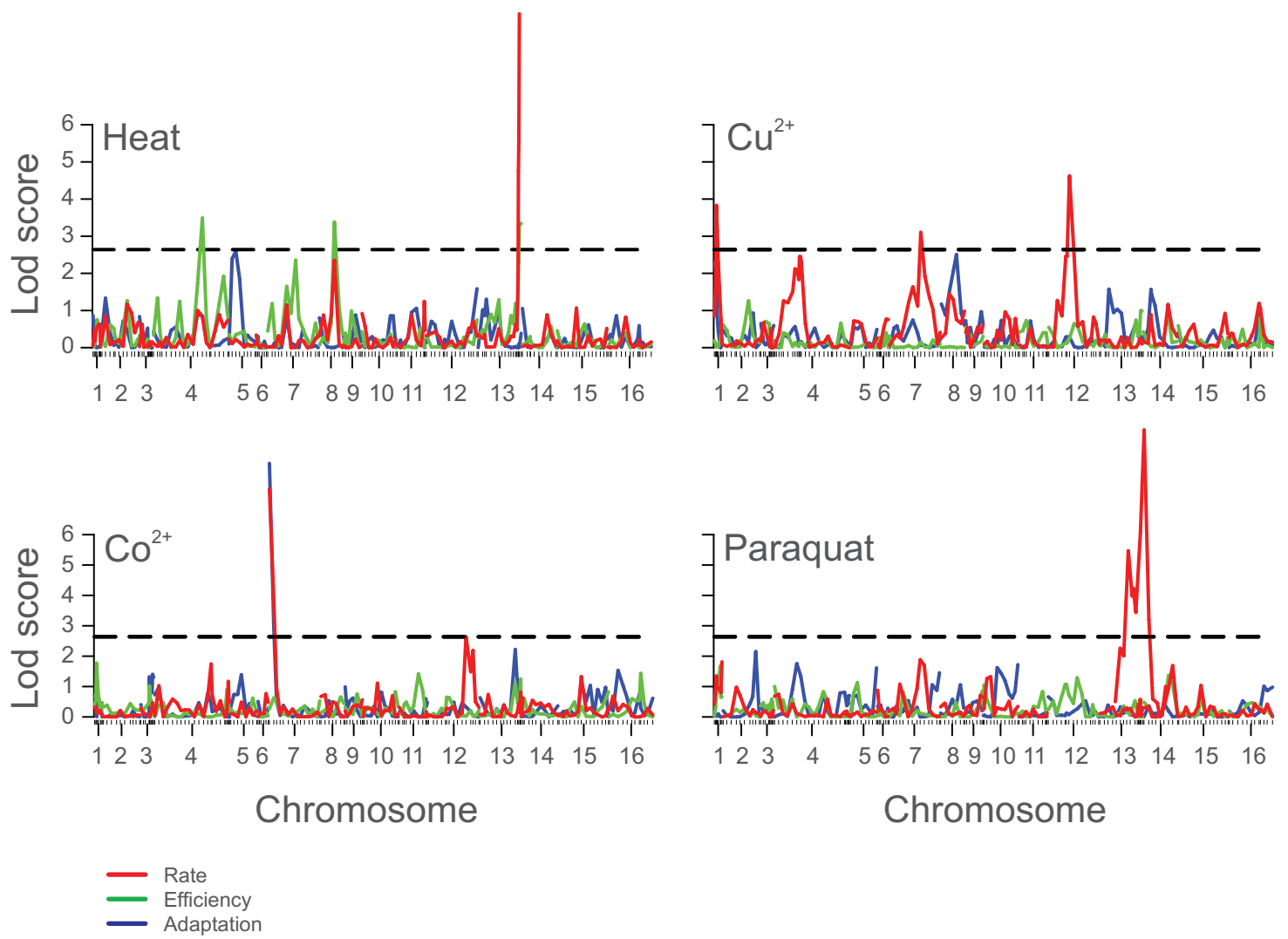

Figure S13 Warringer *et al*

Supplement: Figure S13 — Low performance traits of the West African population map to different QTLs. To map the chromosomal location of the many phenotypes of the West African phenotypic burst, the West African DBVPG6044 was crossed to the North American YPS128, the Sake Y12, and the European DBVPG6765 as described [26]. For each cross, 96 haploid F1 offspring were obtained after meiotic recombination, and co-inheritance of the West African DBVPG6044 proliferation defects and 130 chromosomal markers was investigated using linkage analysis. Figures depict LOD score plots for the co-inheritance of each chromosomal marker and four West African traits: low heat tolerance (the cross DBVPG6044×YPS128), low copper tolerance (the cross DBVPG6044×DBVPG6765), low cobalt tolerance and low paraquat tolerance (both in the cross DBVPG6044×Y12). Chromosome numbers indicate centromere position on each chromosome and tick marks indicate the position of each marker. (PDF) [file pgen.1002111.s014.pdf]

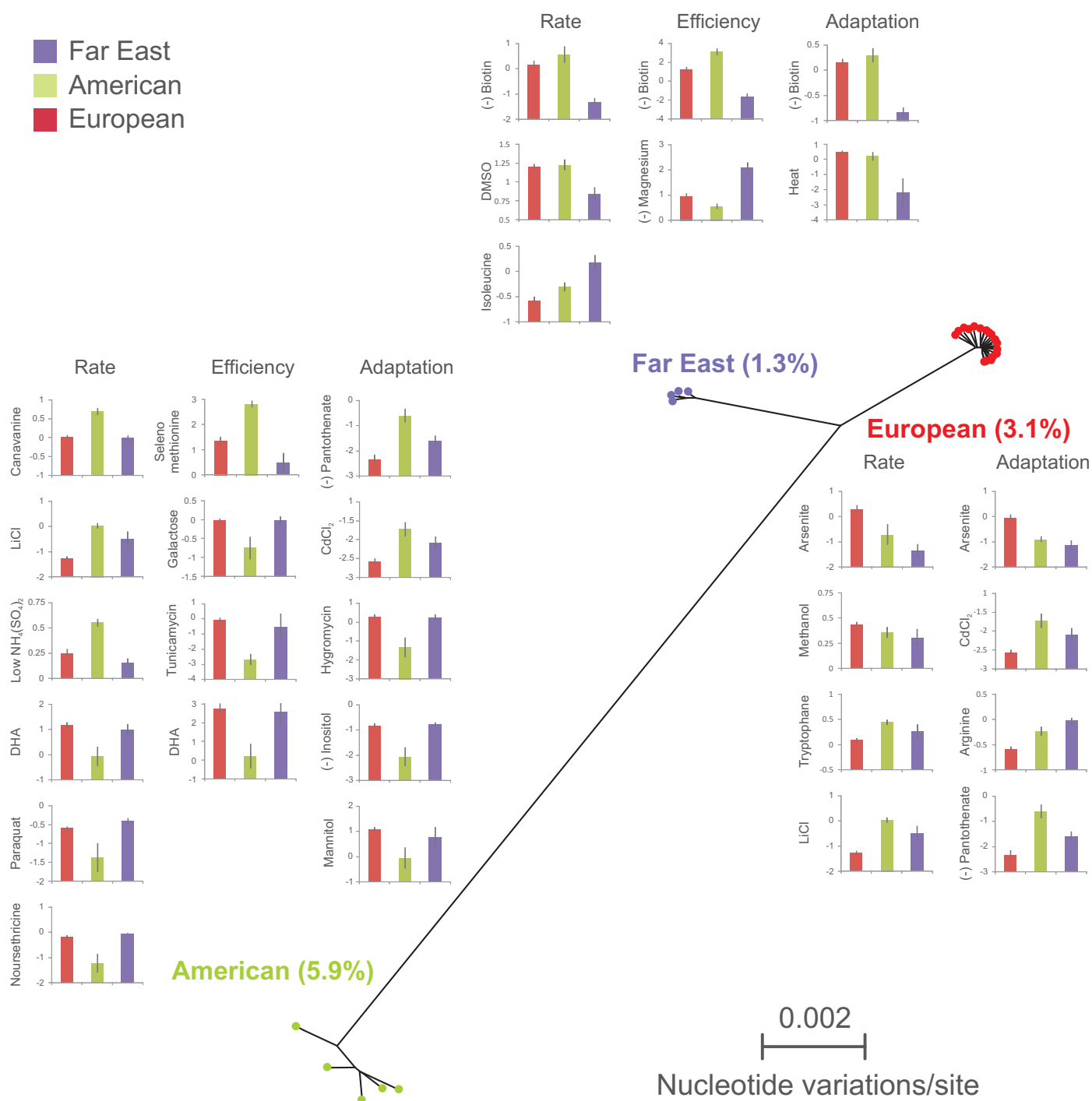

Figure S14 Warringer *et al*

Supplement: Figure S14 — Reconstructing the phenotypic history of S. paradoxus. Population specific traits in S. paradoxus were mapped onto the S. paradoxus population genomics tree [16]. Population specific traits were defined as traits where the proliferative performance of one population deviated significantly from other S. paradoxus isolates (FDR = 2%). Percentages indicate the frequency of population specific traits in each population. Inset bar diagrams show a subset of population specific traits. Bars represent trait averages with bar color indicating population and error bars representing standard errors. Total number of population specific phenotypes: American = 36, European = 19, Far East = 8. Bar color indicates population, error bars indicate standard errors of population averages. (PDF) [file pgen.1002111.s015.pdf]

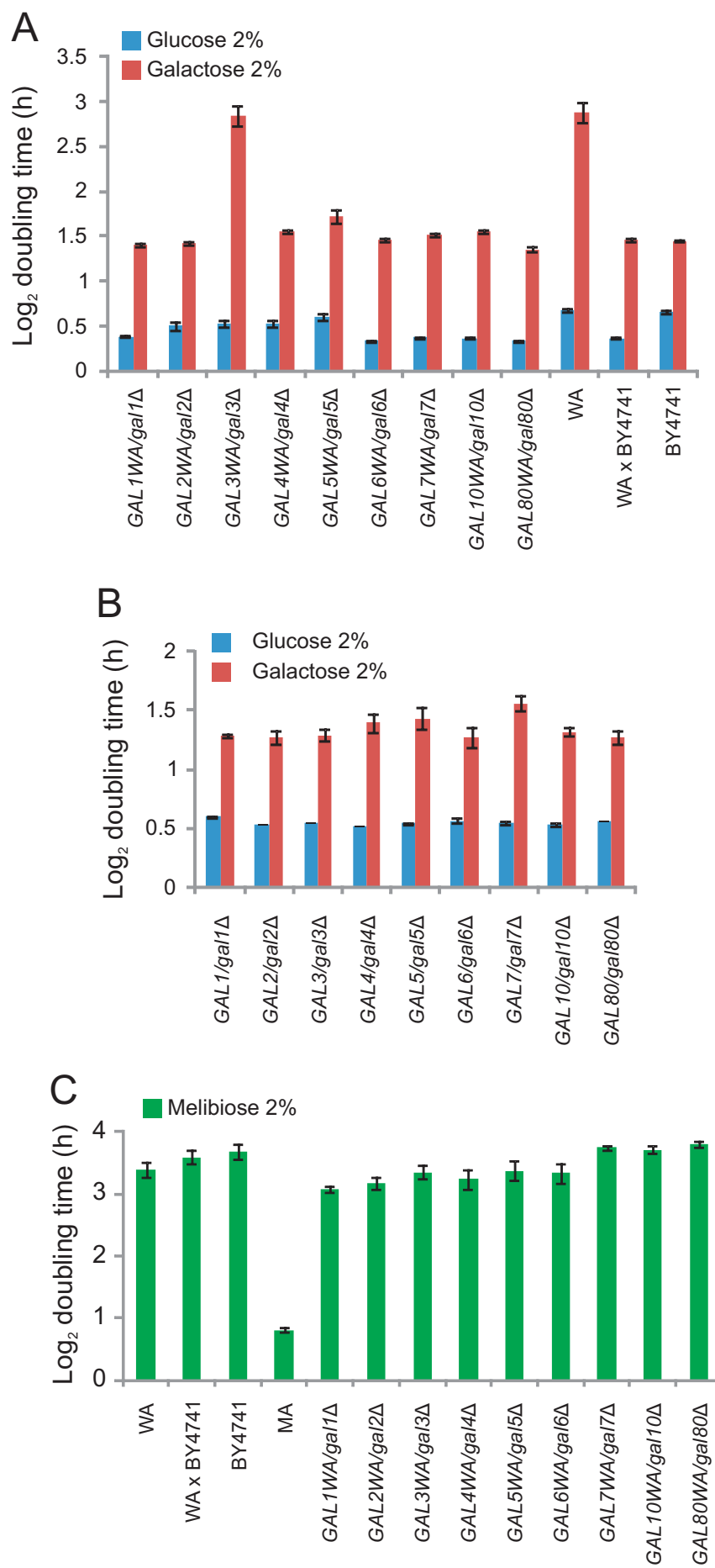

Figure S15 Warringer *et al*

Supplement: Figure S15 — The West African galactose utilization defect is due to a defect in GAL3. A) Deletion strains for all components in the galactose utilization pathway were crossed to the galactose defect West African strain DBVPG6044 (WA) to form diploid hemizygotes, BY4741 (galxΔ)×WA. Hemizygotes as well as their haploid parents and the hybrid BY4741×WA were micro-cultivated for 72 h in glucose and galactose medium respectively and the population doubling time was quantified. Only the BY4741 (gal3Δ)×WA hemizygote shows a galactose growth defect. B) Population doubling time of heterozygote deletion strains (BY4743) corresponding to galactose utilization pathway components, in galactose and glucose respectively. All heterozygotes showed unperturbed growth, including the gal3Δ heterozygote, demonstrating that GAL gene hemizygosity per se does not affect galactose growth. C) Deletion strains for all components in the galactose utilization pathway were crossed to the galactose defect West African strain DBVPG6044 (WA) to form diploid hemizygotes, BY4741 (galxΔ)×WA. Hemizygotes as well as their haploid parents and the hybrid BY4741×WA were micro-cultivated for 72 h in glucose and galactose medium respectively and the population doubling time was quantified. The Malaysian strain UWOPS05_217_3 is shown for comparison. The WA melibiose utilization defect is not suppressed by presence of any of the BY GAL genes. (PDF) [file pgen.1002111.s016.pdf]

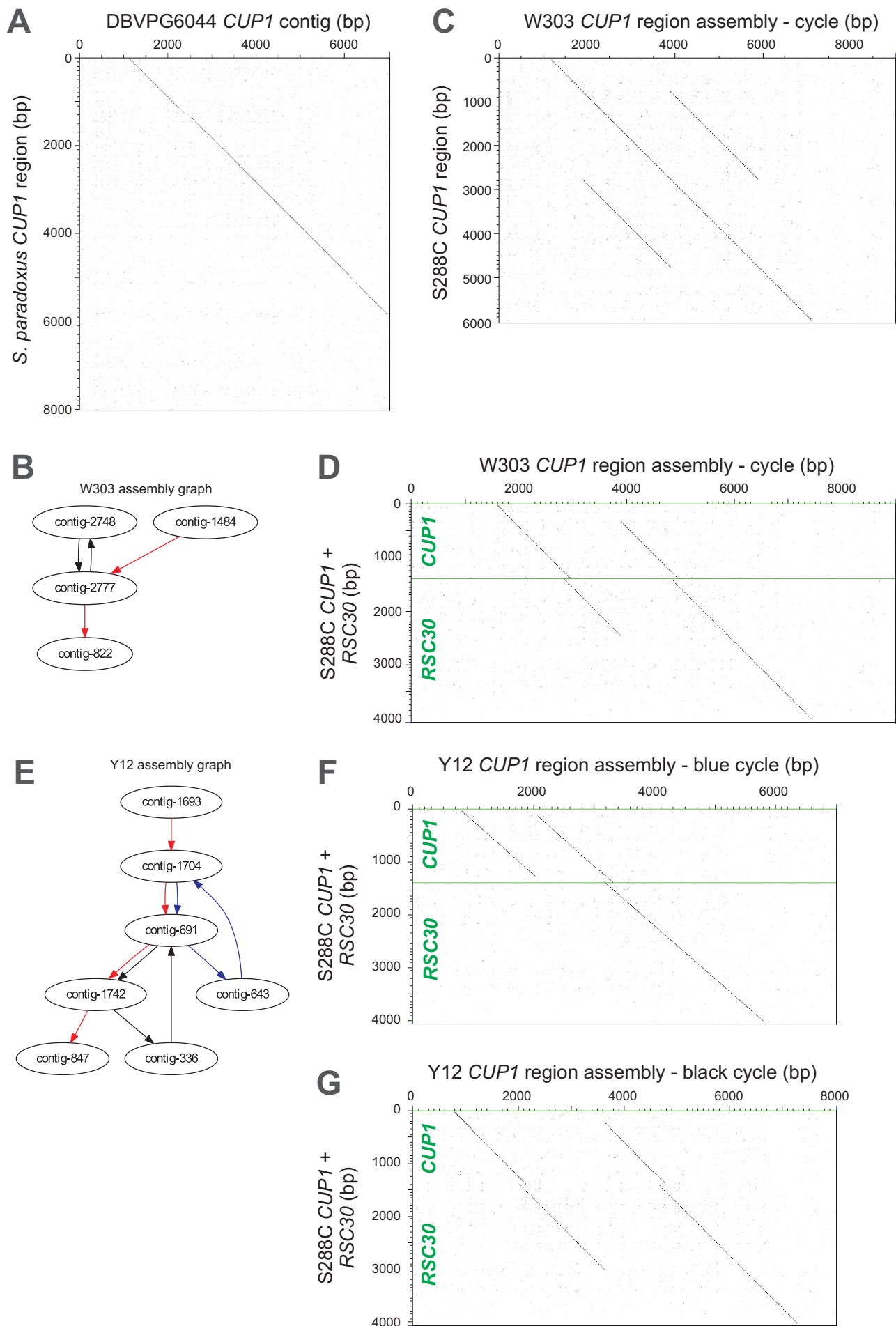

Figure S16 Warringer *et al*

Supplement: Figure S16 — Independent amplifications of the CUP1 locus in the S. cerevisiae Sake and European populations. To test the hypothesis of independent amplifications of CUP1 in the Sake isolate Y12 and the European derived W303, we investigated the breakpoints of the CUP1 amplification in Y12 and W303 by de novo assembly. Y12, W303 and the West African isolate DBVPG6044 was sequenced to 25, 31 and 29-fold coverage respectively with 108 bp reads from a 300 bp insert using the Illumina Genome Analyzer. Reads for each strain were independently assembled using the SGA (string graph assembly) algorithm (Simpson and Durbin, in preparation). SGA is a graph-based assembler which derives the relationship between sequence reads using the FM-index data structure [54]. The graph is traversed to find unambiguous walks which are output as sequence contigs and the topology of the graph indicates the presence and structure of amplified sequence. To identify CUP1 amplification breakpoints, the structure of the assembly graph around the CUP1 locus was analyzed for each strain separately. We mapped the sequence of CUP1 to each assembly to identify matching contigs. A) Dotplot of the CUP1 contig in DBVPG6044 vs. the CUP1 region in S. paradoxus. In DBVPG6044, CUP1 is located in a single contig without breaks, strongly indicating that CUP1 is not duplicated in this strain. We compared the sequence of the matched DBVPG6044 contig to the corresponding locus in S. paradoxus, confirming that the layout of the CUP1 locus in DBVPG6044 is identical to that of S. paradoxus and consistent with a single CUP1. B) Assembly graph of CUP1 sequence in W303 contigs. The CUP1 sequence had three partial matches to W303 contigs. The region of the assembly graph containing these contigs was manually inspected and a simple cycle (black arrows) was found, indicating duplication. C) Dotplot of the CUP1 region in W303 as compared to the CUP1 region in the S. cerevisiae reference genome (S288C). To find the breakpoint of the am [file pgen.1002111.s017.pdf]

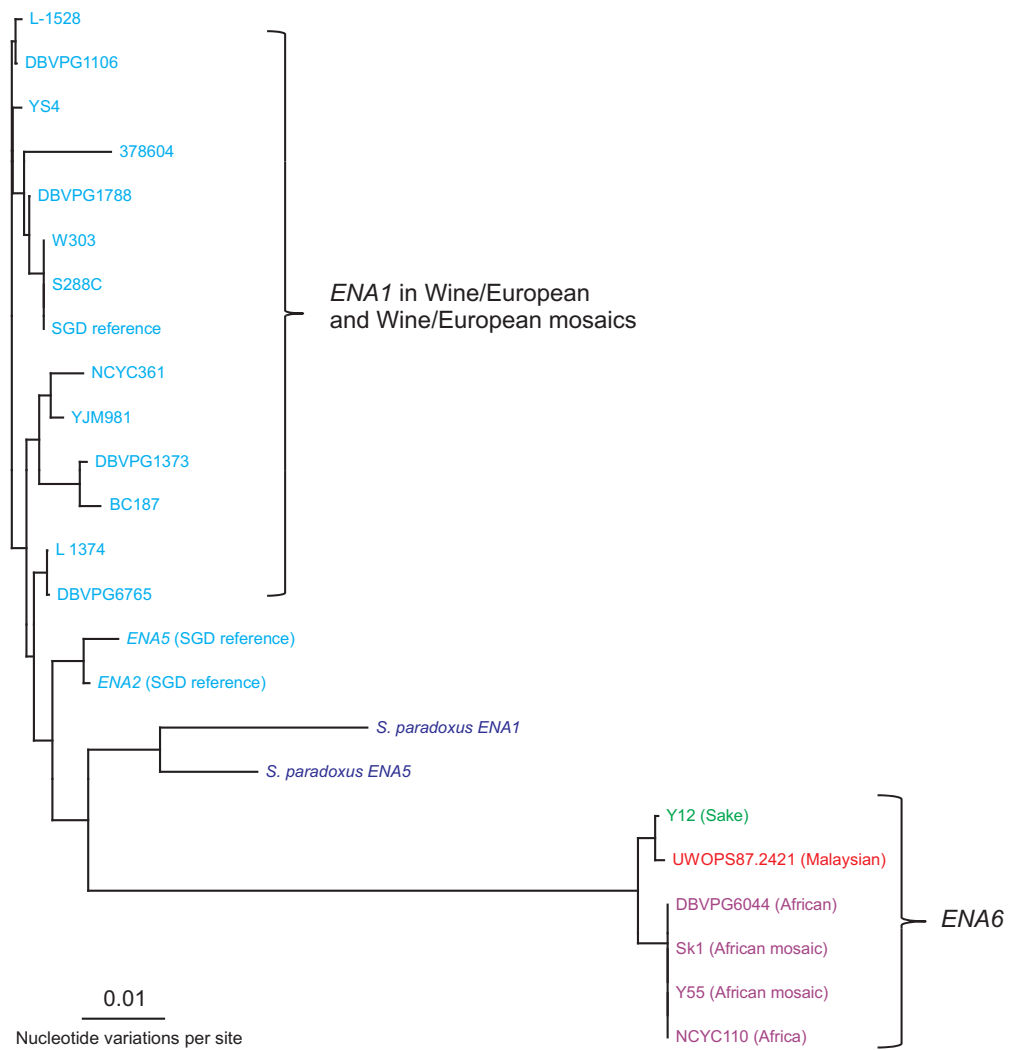

Figure S17 Warringer *et al*

Supplement: Figure S17 — The history of ENA genes in the S. cerevisiae European population diverge from that of the ENA genes in other S. cerevisiae populations. The ENA1 gene sequence from the S. cerevisiae reference genome contained in the Saccharomyces Genome Database (http://www.yeastgenome.org/) was BLASTed (BlastN) against S. cerevisiae genomes [16]. The top hit in each genome was retained and a multiple alignment using ClustalW was performed. The ENA2 and ENA5 reference strain paralogs and the two S. paradoxus ENA orthologs were included for comparison. The ENA genes of the European population and of mosaics predominantly of European origin show an evolutionary history that diverges from that of the ENA gene (ENA6) in the other populations. An ENA6 ortholog was also found in the North American YPS128 and YPS606; however, sequences were fragmented and a high confidence assembly could not be made. (PDF) [file pgen.1002111.s018.pdf]

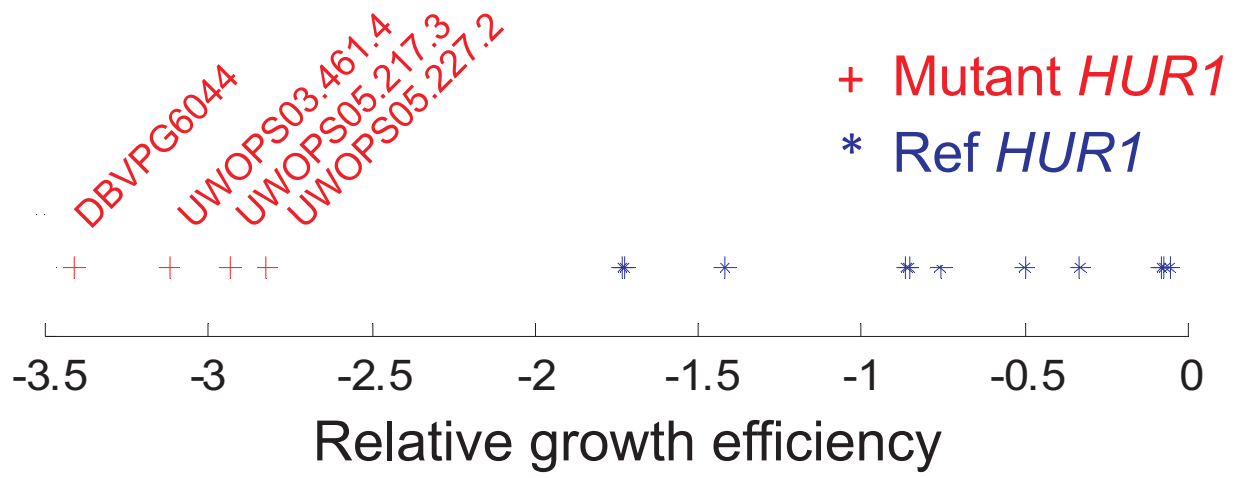

Figure S18 Warringer *et al*

Supplement: Figure S18 — A stop codon in HUR1 explains low tolerance to hydroxyurea in West African and Malaysian S. cerevisiae populations. A premature AAA→TAA (K→Stop) stop codon in HUR1 shows significant association (Student's t-test p<0.015, Kolmygorov-Smirnov p<0.15) to the proliferative efficiency during exposure to 15 mg/mL hydroxyurea. West African and Malaysian derived genomes (DBVPG6044, UWOPS03.461.4, UWOPS05.217.3, UWOPS05.227.2) contain the variant mutation, 14 other strains contain the reference sequence. (PDF) [file pgen.1002111.s019.pdf]
