# Supplementary material for: Trait Variation in Yeast Is Defined by Population History
Source: PLoS Genet. 2011 Jun 16;7(6):e1002111. doi: 10.1371/journal.pgen.1002111 (PMC3116910; doi:10.1371/journal.pgen.1002111)
Supplement: Table S1 — Strains used in the study. ID refers to the strain ID number in the Gianni Liti collection. For further details on S. cerevisiae and S. paradoxus strains, see Liti et al [16]. (DOC) [file pgen.1002111.s020.doc]

**TABLE S1** **Strains used in the study.** ID refers to the strain ID number in the Gianni Liti collection. For further details on *S. cerevisiae* and *S. paradoxus* strains, see Liti *et al* .

| **Name** | **ID** | **Species** | **Population** | **Source** | **Provided by** |
| --- | --- | --- | --- | --- | --- |
| **350** | 350 | *S. arboricolus* | Unknown | Wild |  |
| **351** | 351 | *S. arboricolus* | Unknown | Wild |  |
| **352** | 352 | *S. arboricolus* | Unknown | Wild |  |
| **CBS1001** | 24 | *S. bayanus* | Unknown | Wild |  |
| **274** | 274 | *S. bayanus* | Unknown | Wild |  |
| **CBSG309** | 138 | *S. castelli* | Unknown | Wild |  |
| **S288c** | 96 | *S. cerevisiae* | Mosaic | Lab | Haber JE |
| **Y55** | 97 | *S. cerevisiae* | Mosaic, African | Lab | Haber JE |
| **SK1** | 17 | *S. cerevisiae* | Mosaic, African | Lab | Haber JE |
| **W303** | 281 | *S. cerevisiae* | Mosaic | Lab | EUROFAN |
| **YJM978** | 303 | *S. cerevisiae* | European | Clinical | McCusker J |
| **YJM981** | 304 | *S. cerevisiae* | European | Clinical | McCusker J |
| **YJM975** | 308 | *S. cerevisiae* | European | Clinical | McCusker J |
| **322134S** | 284 | *S. cerevisiae* | Mosaic, European | Clinical | Mackenzie D |
| **378604X** | 287 | *S. cerevisiae* | Mosaic, European | Clinical | Mackenzie D |
| **273614N** | 288 | *S. cerevisiae* | Mosaic, European | Clinical |  |
| **DBVPG1788** | 84 | *S. cerevisiae* | European | Wild | Vaughan A |
| **DBVPG1373** | 91 | *S. cerevisiae* | European | Wild | Vaughan A |
| **YIIc17_E5** | 174 | *S. cerevisiae* | Mosaic, European | Fermentation | Souciet JL |
| **DBVPG6040** | 155 | *S. cerevisiae* | Mosaic, European | Fermentation | Vaughan A |
| **NCYC361** | 248 | *S. cerevisiae* | Mosaic, European | Fermentation | NCYC |
| **YPS606** | 182 | *S. cerevisiae* | North American | Wild | Gerke J |
| **YPS128** | 104 | *S. cerevisiae* | North American | Wild | Sniegowski P |
| **YS2** | 258 | *S. cerevisiae* | Mosaic, european | Baking | Bell P |
| **YS4** | 259 | *S. cerevisiae* | Mosaic, European | Baking | Bell P |
| **YS9** | 262 | *S. cerevisiae* | Mosaic, European | Baking | Bell P |
| **UWOPS03-461.4** | 278 | *S. cerevisiae* | Malaysia | Wild | Lachance M |
| **UWOPS05-217.3** | 279 | *S. cerevisiae* | Malaysia | Wild | Lachance M |
| **UWOPS05-227.2** | 280 | *S. cerevisiae* | Malaysia | Wild | Lachance M |
| **K11** | 251 | *S. cerevisiae* | Sake | Fermentation |  |
| **Y9** | 252 | *S. cerevisiae* | Sake | Fermentation |  |
| **RM11** | 345 | *S. cerevisiae* | European | Fermentation |  |
| **DBVPG1853** | 92 | *S. cerevisiae* | Mosaic, European | Wild | Vaughan A |
| **Y12** | 253 | *S. cerevisiae* | Sake | Fermentation |  |
| **NCYC110** | 247 | *S. cerevisiae* | Africa | Fermentation | NCYC |
| **DBVPG6044** | 60 | *S. cerevisiae* | Africa | Fermentation | Vaughan A |
| **DBVPG6765** | 3 | *S. cerevisiae* | European | Unknown | Vaughan A |
| **L-1374** | 220 | *S. cerevisiae* | European | Fermentation | Martinez C |
| **L-1528** | 221 | *S. cerevisiae* | European | Fermentation | Martinez C |
| **UWOPS87-2421** | 271 | *S. cerevisiae* | Mosaic | Wild | Lachance M |
| **DBVPG1106** | 150 | *S. cerevisiae* | European | Fermentation | Vaughan A |
| **UWOPS83-787.3** | 270 | *S. cerevisiae* | Mosaic | Wild | Lachance M |
| **YJM789** | 238 | *S. cerevisiae* | Mosaic, European | Clinical |  |
| **BC187** | 181 | *S. cerevisiae* | European | Fermentation | Gerke J |
| **1802** | 22 | *S. kudriavzevii* |  | Wild |  |
| **1803** | 23 | *S. kudriavzevii* |  | Wild |  |
| **IFO1815** | 18 | *S. mikatae* |  | Wild |  |
| **IFO1816** | 19 | *S. mikatae* |  | Wild |  |
| **CBS432** | 142 | *S. paradoxus* | European |  | Naumov G |
| **N-17** | 26 | *S. paradoxus* | European |  | Naumov G |
| **CBS5829** | 98 | *S. paradoxus* | European |  | Naumov G |
| **DBVPG4650** | 28 | *S. paradoxus* | European |  | Vaughan A |
| **T21.4** | 40 | *S. paradoxus* | European |  | Koufopanou V |
| **Y7** | 164 | *S. paradoxus* | European |  | Koufopanou V |
| **Y6.5** | 165 | *S. paradoxus* | European |  | Koufopanou V |
| **Q32.3** | 167 | *S. paradoxus* | European |  | Koufopanou V |
| **Q59.1** | 168 | *S. paradoxus* | European |  | Koufopanou V |
| **Q62.5** | 169 | *S. paradoxus* | European |  | Koufopanou V |
| **Q89.8** | 170 | *S. paradoxus* | European |  | Koufopanou V |
| **Q95.3** | 171 | *S. paradoxus* | European |  | Koufopanou V |
| **S36.7** | 172 | *S. paradoxus* | European |  | Koufopanou V |
| **Z1.1** | 173 | *S. paradoxus* | European |  | Koufopanou V |
| **Y9.6** | 293 | *S. paradoxus* | European |  | Koufopanou V |
| **379** | 379 | *S. paradoxus* | European |  |  |
| **Q74.4** | 294 | *S. paradoxus* | European |  | Koufopanou V |
| **Q96.8** | 296 | *S. paradoxus* | European |  |  |
| **LD7** | 297 | *S. paradoxus* | European |  |  |
| **Q31.4** | 298 | *S. paradoxus* | European |  | Koufopanou V |
| **Y8.5** | 299 | *S. paradoxus* | European |  | Koufopanou V |
| **Z1** | 301 | *S. paradoxus* | European |  | Koufopanou V |
| **Y8.1** | 302 | *S. paradoxus* | European |  | Koufopanou V |
| **KPN3828** | 254 | *S. paradoxus* | European |  | Iurkow A |
| **KPN3829** | 255 | *S. paradoxus* | European |  | Iurkow A |
| **YPS138** | 115 | *S. paradoxus* | American |  | Sniegowski P |
| **DBVPG6304** | 32 | *S. paradoxus* | American |  | Vaughan A |
| **A4** | 186 | *S. paradoxus* | American |  | Koufopanou V |
| **A12** | 187 | *S. paradoxus* | American |  | Koufopanou V |
| **UFRJ50791** | 20 | *S. paradoxus* | American |  | Naumov G |
| **UFRJ50816** | 21 | *S. paradoxus* | American |  | Naumov G |
| **N-43** | 76 | *S. paradoxus* | Far East |  | Naumov G |
| **N-44** | 77 | *S. paradoxus* | Far East |  | Naumov G |
| **N-45** | 78 | *S. paradoxus* | Far East |  | Naumov G |
| **IFO1804** | 137 | *S. paradoxus* | Far East |  | Pérez-Ortín J |
| **388** | 388 | *S. bayanus* |  |  |  |
| **391** | 391 | *S. kudriavzevii* |  |  |  |
| **392** | 392 | *S. kudriavzevii* |  |  |  |
