# Supplementary material for: Trait Variation in Yeast Is Defined by Population History
Source: PLoS Genet. 2011 Jun 16;7(6):e1002111. doi: 10.1371/journal.pgen.1002111 (PMC3116910; doi:10.1371/journal.pgen.1002111)
Supplement: Table S2 — Environments used in the screen. The classification “carbon utilization” indicates that 2% glucose in these experiments was substituted with the indicated amounts of the indicated carbon source, the classification “nitrogen utilization” indicates that 0.5% ammonium sulfate was substituted with the indicated nitrogen sources at nitrogen limiting concentrations (see Text S1). # = two consecutive pre-cultures were performed, the first with nitrogen limiting amounts of ammonium sulfate (low ammonium sulfate), the second with nitrogen limiting amounts of the indicated nitrogen source. * = pre-cultures were performed in medium identical to the experimental medium. (DOC) [file pgen.1002111.s021.doc]

**Table S2 Environments used in the screen. The classification ”carbon utilization” indicates that 2% glucose in these experiments was substituted with the indicated amounts of the indicated carbon source, the classification “nitrogen utilization” indicates that 0.5% ammonium sulfate was substituted with the indicated nitrogen sources at nitrogen limiting concentrations (see Supplementary Materials and Methods). # = two consecutive pre-cultures were performed, the first with nitrogen limiting amounts of ammonium sulfate (low ammonium sulfate), the second with nitrogen limiting amounts of the indicated nitrogen source. * = pre-cultures were performed in medium identical to the experimental medium.**

| **Environment** | **Class** |
| --- | --- |
| Ethanol 2% | Carbon utilization |
| Fructose 2% | Carbon utilization |
| Galactose 2% | Carbon utilization |
| Glucose 0.5% | Carbon utilization |
| Glucose 1% | Carbon utilization |
| Glucose 16% | Carbon utilization |
| Glucose 2% | Carbon utilization |
| Glucose 32% | Carbon utilization |
| Glucose 4% | Carbon utilization |
| Glucose 8% | Carbon utilization |
| Glycerol 2% | Carbon utilization |
| Lactose 2% | Carbon utilization |
| Maltose 2% | Carbon utilization |
| Maltose 8% | Carbon utilization |
| Mannitol 2% | Carbon utilization |
| Mannose 2% | Carbon utilization |
| Melezitose 2% | Carbon utilization |
| Melibiose 2% | Carbon utilization |
| Methanol 2% | Carbon utilization |
| Raffinose 2% | Carbon utilization |
| Sorbitol 2% | Carbon utilization |
| Starch 1% | Carbon utilization |
| Sucrose 2% | Carbon utilization |
| Trehalose 2% | Carbon utilization |
| Xylitol 2% | Carbon utilization |
| 1,1-Phenanthroline 2µM | Toxins |
| 1,1-Phenantroline 4µM | Toxins |
| 1,2,4-Aminotriazole 300mM | Toxins |
| 124-Aminotriazole 450 mM | Toxins |
| 6-azauracil 200µg/mL | Toxins |
| 6-azauracil 300µg/mL | Toxins |
| 6-azauracil 500µg/mL | Toxins |
| Anisomycin 0.5µg/mL | Toxins |
| Anisomycin 1µg/mL | Toxins |
| Arabinose 2% | Toxins |
| Caffeine 1.5mg/mL | Toxins |
| Caffeine 2.25mg/mL | Toxins |
| Canavanine 100µg/mL | Toxins |
| Canavanine 2µg/mL | Toxins |
| Canavanine 3µg/mL | Toxins |
| Cisplatin 100µg/mL | Toxins |
| Cisplatin 150µg/mL | Toxins |
| Clotrimazole 1.5µM | Toxins |
| Clotrimazole 3µM | Toxins |
| Cykloheximide 0.05µg/mL | Toxins |
| Cykloheximide 0.1µg/mL | Toxins |
| DMSO 0.25% | Toxins |
| DMSO 2% | Toxins |
| DMSO 4% | Toxins |
| DMSO 8% | Toxins |
| DTT 1.6mM | Toxins |
| DTT 1.8mM | Toxins |
| Geneticin 2mM | Toxins |
| Geneticin 3mM | Toxins |
| Geneticin 4mM | Toxins |
| Hog1 inhibitor TA47:2 2µM | Toxins |
| Hog1 inhibitor TA47:2 8uM | Toxins |
| Hydroxyurea 10mg/mL | Toxins |
| Hydroxyurea 15mg/mL | Toxins |
| Hygromycin 0.6mg/mL | Toxins |
| Hygromycin B 1.25mg/mL | Toxins |
| Mercaptoethanol 15mM | Toxins |
| Mercaptoethanol 30mM | Toxins |
| MMS 0.005% | Toxins |
| MMS 0.01% | Toxins |
| Noursethricine 10µg/mL | Toxins |
| Noursethricine 100µg/mL | Toxins |
| Noursethricine 5µg/mL | Toxins |
| Paramomycin 10mg/mL | Toxins |
| Paramomycin 15mg/mL | Toxins |
| Paraquat 250µg/mL | Toxins |
| Paraquat 400µg/mL | Toxins |
| Rapamycin 0.5µg/mL | Toxins |
| Rapamycin 1µg/mL | Toxins |
| Sodium arsenite oxide 3.5mM | Toxins |
| Sodium arsenite oxide 5mM | Toxins |
| Staurosporine 10ug/mL | Toxins |
| Staurosporine 1ug/mL | Toxins |
| Thiabendazole 25µg/mL | Toxins |
| Thiabendazole 50µg/mL | Toxins |
| Trifluoperazine 100µM | Toxins |
| Trifluoperazine 50µM | Toxins |
| Tunicamycin 1.5µg/mL | Toxins |
| Tunicamycin 1µg/mL | Toxins |
| CdCl_2_ 100µM | Environment & Metabolites |
| CdCl_2_ 150µM | Environment & Metabolites |
| Citric acid 46mg/mL | Environment & Metabolites |
| Citric acid 55mg/mL | Environment & Metabolites |
| Citric acid 62.5mg/mL | Environment & Metabolites |
| CoCl_2_ 0.015mM | Environment & Metabolites |
| CoCl_2_ 0.01mM | Environment & Metabolites |
| Cold 16°C | Environment & Metabolites |
| Cold 19°C | Environment & Metabolites |
| Cold 24°C | Environment & Metabolites |
| CuCl_2_ 0.375mM | Environment & Metabolites |
| CuCl_2_ 0.75mM | Environment & Metabolites |
| DHA 100mM | Environment & Metabolites |
| DHA 200mM | Environment & Metabolites |
| Ethanol 13% | Environment & Metabolites |
| Ethanol 16% | Environment & Metabolites |
| Ethanol 5% | Environment & Metabolites |
| Ethanol 7% | Environment & Metabolites |
| Formaldehyde 0.4mM | Environment & Metabolites |
| Formaldehyde 1mM | Environment & Metabolites |
| Formaldehyde 2mM | Environment & Metabolites |
| Heat 37°C | Environment & Metabolites |
| Heat 40°C | Environment & Metabolites |
| Heat 41°C | Environment & Metabolites |
| Heat 42°C | Environment & Metabolites |
| Heat 43°C | Environment & Metabolites |
| KCl 1.45M | Environment & Metabolites |
| KCl 2M | Environment & Metabolites |
| LiCl 150mM | Environment & Metabolites |
| LiCl 225mM | Environment & Metabolites |
| Malic acid 52mg/mL | Environment & Metabolites |
| Malic acid 62.5mg/mL | Environment & Metabolites |
| Methanol 10% | Environment & Metabolites |
| Methanol 2% | Environment & Metabolites |
| Methanol 5% | Environment & Metabolites |
| Methanol 7.5% | Environment & Metabolites |
| Methylglyoxal 2mM | Environment & Metabolites |
| Methylglyoxal 4mM | Environment & Metabolites |
| NaCl 0.85M | Environment & Metabolites |
| NaCl 1.25M | Environment & Metabolites |
| NiCl_2_ 0.375mM | Environment & Metabolites |
| NiCl_2_ 0.75mM | Environment & Metabolites |
| Oxalic acid 12.5mg/mL | Environment & Metabolites |
| Oxalic acid 8mg/mL | Environment & Metabolites |
| pH 2 | Environment & Metabolites |
| pH 3.5 | Environment & Metabolites |
| pH 7 | Environment & Metabolites |
| Selenomethionine 0.03mM | Environment & Metabolites |
| Selenomethionine 0.06mM | Environment & Metabolites |
| Synthetic wine must 2% | Environment & Metabolites |
| Tartaric acid 45mg/mL | Environment & Metabolites |
| Tartaric acid 60mg/mL | Environment & Metabolites |
| YPD | Environment & Metabolites |
| Adenine # | Nitrogen utilization |
| Alanine # | Nitrogen utilization |
| Allantoine # | Nitrogen utilization |
| Arginine # | Nitrogen utilization |
| Asparagine # | Nitrogen utilization |
| Aspartic acid # | Nitrogen utilization |
| Citrulline # | Nitrogen utilization |
| Cytidine # | Nitrogen utilization |
| Cytosine # | Nitrogen utilization |
| GABA # | Nitrogen utilization |
| Glutathione # | Nitrogen utilization |
| Glycine # | Nitrogen utilization |
| Guanidine # | Nitrogen utilization |
| Histidine # | Nitrogen utilization |
| Isoleucine # | Nitrogen utilization |
| Leucine # | Nitrogen utilization |
| Low ammonium sulfate * | Nitrogen utilization |
| Lysine # | Nitrogen utilization |
| Methionine # | Nitrogen utilization |
| Ornithine # | Nitrogen utilization |
| Phenylalanine # | Nitrogen utilization |
| Proline # | Nitrogen utilization |
| Serine # | Nitrogen utilization |
| Threonine # | Nitrogen utilization |
| Tryptophane # | Nitrogen utilization |
| Tyrosine # | Nitrogen utilization |
| Uracil # | Nitrogen utilization |
| Urea # | Nitrogen utilization |
| Valine # | Nitrogen utilization |
| Biotin depletion | Nutrient requirements |
| Biotin depletion * | Nutrient requirements |
| Boron (B) depletion * | Nutrient requirements |
| Ca depletion | Nutrient requirements |
| Ca depletion * | Nutrient requirements |
| Cu depletion | Nutrient requirements |
| Inositol depletion | Nutrient requirements |
| Inositol depletion * | Nutrient requirements |
| Fe depletion * | Nutrient requirements |
| Adenine depletion | Nutrient requirements |
| No aminoacids * | Nutrient requirements |
| Mb depletion * | Nutrient requirements |
| Leucine depletion | Nutrient requirements |
| Lysine depletion | Nutrient requirements |
| Mg depletion | Nutrient requirements |
| Mg depletion * | Nutrient requirements |
| Mn depletion * | Nutrient requirements |
| Methionine depletion | Nutrient requirements |
| Na depletion * | Nutrient requirements |
| Nicotinamide depletion | Nutrient requirements |
| Nicotinamide depletion | Nutrient requirements |
| PABA depletion | Nutrient requirements |
| PABA depletion * | Nutrient requirements |
| Pantothenate depletion | Nutrient requirements |
| Pantothenate depletion * | Nutrient requirements |
| Phosphate depletion | Nutrient requirements |
| Phosphate depletion * | Nutrient requirements |
| Pyridoxin depletion * | Nutrient requirements |
| Riboflavin depletion * | Nutrient requirements |
| Thiamine depletion | Nutrient requirements |
| Thiamine depletion * | Nutrient requirements |
| Tryptophane depletion | Nutrient requirements |
| Uracil depletion | Nutrient requirements |
| Zinc depletion * | Nutrient requirements |
