# Supplementary material for: Trait Variation in Yeast Is Defined by Population History
Source: PLoS Genet. 2011 Jun 16;7(6):e1002111. doi: 10.1371/journal.pgen.1002111 (PMC3116910; doi:10.1371/journal.pgen.1002111)
Supplement: Table S4 — Nonsense mutations in natural yeast isolates. Premature stop codon mutations with a minor allele frequency ≥3 in S. cerevisiae. Stop codons in dubious genes, as well as in genes with a high number of premature stop codons (>2), were not included. In all cases, the reference sequence contains an amino acid encoding codon whereas the variation is a stop codon mutation. Strains carrying a premature stop codon are represented by “0”, whereas strains carrying the reference sequence are represented by “1”. Empty cells correspond to missing data for the associated strain. (DOC) [file pgen.1002111.s023.doc]

**Table S4 Nonsense mutations in natural yeast isolates**

Stop codon mutations with a minor allele frequency ≥3 in *S. cerevisiae*. Stop codons in dubious genes, as well as in genes with a high number of stop codons (>2), are not included. In all cases, the reference sequence contains an amino acid encoding codon whereas the variation is a stop codon mutation. Strains carrying a premature stop codon are represented by “0”, whereas strains carrying the reference sequence are represented by “1”. Empty cells correspond to missing data for the associated strain.

| Name | Stop position (a.a.) | protein length | Wine/European | | | | | | | | | | | | NA | | | MA | | | | Sake | | | | WA | | | Mosaics | | | | | | | | | | | | | | | | |  |
| --- | --- | --- | --- | --- | --- | --- | --- | --- | --- | --- | --- | --- | --- | --- | --- | --- | --- | --- | --- | --- | --- | --- | --- | --- | --- | --- | --- | --- | --- | --- | --- | --- | --- | --- | --- | --- | --- | --- | --- | --- | --- | --- | --- | --- | --- | --- |
|  |  |  | RM11_1A | L_1374 | L_1528 | BC187 | YJM975 | YJM978 | YJM981 | DBVPG1106 | DBVPG1373 | DBVPG1788 | DBVPG1853 | DBVPG6765 | | YPS128 | YPS606 | | UWOPS03_461_4 | UWOPS05_217_3 | UWOPS05_227_2 | | Y12 | Y9 | K11 | | DBVPG6044 | NCYC110 | | SK1 | Y55 | NCYC361 | S288c | W303 | DBVPG6040 | UWOPS83_787_3 | UWOPS87_2421 | 273614N | 322134S | 378604X | YIIc17_E5 | YJM789 | YS2 | YS4 | YS9 | |
| AQR1 | 562 | 586 | 0 |  |  | 0 | 0 |  |  | 0 | 0 | 0 |  | 0 | | 0 | 0 | |  |  | 0 | |  |  |  | |  | 0 | | 0 | 0 |  | 1 | 1 |  |  |  | 0 |  |  |  | 0 |  | 0 | 0 | |
| ECM1 | 5 | 212 | 0 |  | 0 |  |  |  |  | 0 | 0 |  | 0 | 0 | | 0 | 0 | | 0 |  |  | |  |  |  | |  | 0 | | 0 | 0 |  | 1 | 1 | 0 |  |  |  |  |  |  | 0 |  | 0 | 0 | |
| ECM12 | 8 | 151 | 1 |  |  |  | 1 | 1 |  |  |  | 1 |  | 1 | |  | 0 | | 1 |  |  | | 0 |  | 0 | |  |  | | 1 | 1 |  |  | 1 |  | 1 | 1 |  | 1 |  |  | 0 | 1 |  | 1 | |
| GAL3 | 151 | 520 | 1 | 1 |  | 1 | 1 |  | 1 | 1 |  |  |  | 1 | | 1 |  | | 1 |  |  | | 1 | 1 | 1 | | 0 | 0 | | 0 | 0 |  | 1 | 1 |  |  |  |  | 1 | 1 | 1 | 1 |  | 1 | 1 | |
| HMS1 | 384 | 434 | 0 |  |  | 0 | 1 |  |  |  |  | 1 |  | 1 | | 1 |  | |  |  |  | | 1 | 1 |  | | 1 | 1 | | 1 | 1 |  | 1 | 1 | 1 |  |  |  |  | 0 | 1 | 1 |  | 1 | 1 | |
| HUR1 | 55 | 110 | 1 |  |  |  |  |  |  |  |  | 1 | 1 | 1 | | 1 | 1 | | 0 | 0 | 0 | |  | 1 | 1 | | 0 |  | | 1 | 1 |  | 1 | 1 |  |  |  |  |  |  |  | 1 |  |  |  | |
| HVG1 | 87 | 249 | 1 |  | 1 | 1 |  |  |  |  |  | 1 |  |  | |  | 1 | | 1 |  |  | |  |  | 1 | | 0 |  | | 0 | 0 |  | 1 | 1 | 1 |  | 1 |  |  |  |  | 1 | 1 |  | 1 | |
| MMP1 | 178 | 583 | 1 |  | 0 | 1 | 1 |  |  |  | 1 |  |  | 0 | | 1 |  | | 1 |  | 1 | | 1 |  |  | | 1 | 1 | | 1 | 1 |  | 1 | 1 |  |  |  | 0 | 0 |  |  |  |  |  | 1 | |
| PAU21 | 17 | 164 |  | 1 | 1 | 1 | 1 | 1 | 1 | 1 | 1 | 1 | 1 | 1 | |  | 0 | | 1 | 1 | 1 | | 0 |  |  | | 0 |  | | 0 | 1 |  | 1 |  | 1 |  | 1 |  |  |  |  |  |  |  |  | |
| SPH1 | 648 | 661 | 0 |  |  |  |  |  |  |  | 0 |  |  | 0 | | 0 | 0 | |  | 0 |  | |  |  |  | |  |  | | 0 | 0 |  | 1 | 1 |  |  | 0 | 0 |  |  |  | 0 |  |  |  | |
| TRM2 | 31 | 639 | 0 |  | 0 |  |  |  |  | 0 | 0 |  |  | 0 | | 0 | 0 | |  |  | 0 | | 0 | 0 |  | | 0 | 0 | | 0 | 0 |  | 1 | 0 | 1 | 0 |  | 1 |  | 1 | 0 | 0 |  |  | 0 | |
| YAR028W | 140 | 234 |  |  | 1 |  |  | 0 |  |  |  | 0 |  | 1 | |  |  | |  | 1 |  | |  |  |  | |  |  | | 1 | 1 |  | 1 | 1 |  |  |  |  | 1 | 1 |  |  |  | 0 | 0 | |
| YBL008W-A | 66 | 79 | 1 | 1 |  |  | 1 |  |  |  |  |  | 1 | 1 | | 1 |  | |  | 1 |  | | 1 |  |  | | 0 |  | | 0 | 0 |  | 1 | 1 |  |  |  | 1 |  |  |  | 1 | 1 |  |  | |
| YCL049C | 136 | 312 | 1 |  | 0 | 1 | 1 | 1 |  |  | 1 |  |  | 0 | | 1 | 1 | | 1 |  |  | |  | 1 | 1 | | 1 |  | | 1 | 1 |  | 1 | 1 |  | 1 |  |  |  | 1 |  | 0 |  |  | 0 | |
| YCL073C | 24 | 615 | 1 | 1 |  |  | 0 |  | 0 |  |  |  |  | 0 | |  | 1 | | 1 | 1 | 1 | |  | 1 |  | |  |  | |  | 0 |  |  |  |  |  |  | 0 |  |  |  |  |  |  |  | |
| YDR114C | 95 | 100 | 1 | 1 |  |  |  |  | 1 |  | 1 |  |  | 1 | | 1 |  | | 1 |  | 1 | | 0 |  | 1 | | 1 |  | | 1 | 1 |  |  |  |  |  | 1 |  | 1 | 1 | 1 | 0 |  | 0 |  | |
| YDR119W | 758 | 768 | 1 |  | 1 | 1 | 1 | 1 |  |  |  | 1 |  | 1 | |  | 0 | |  |  |  | |  |  | 0 | | 0 |  | | 0 | 0 |  | 1 | 1 |  | 0 |  |  | 1 | 1 |  | 0 |  |  |  | |
| YEL067C | 154 | 195 | 0 | 0 |  |  |  |  |  |  | 0 | 0 |  | 0 | |  | 0 | |  |  | 0 | |  |  |  | |  |  | | 0 | 0 |  | 1 | 1 | 1 | 0 |  | 0 |  | 0 |  | 0 |  |  | 1 | |
| YLR408C | 10 | 122 | 1 | 1 |  |  |  | 1 |  |  | 1 |  |  |  | | 0 |  | |  | 0 |  | |  | 0 |  | | 0 | 0 | | 0 | 1 |  | 1 | 1 |  |  | 1 |  |  |  |  | 0 |  |  |  | |
| YOL038C-A | 15 | 31 | 1 |  | 1 |  |  | 1 |  |  |  |  |  | 1 | |  |  | |  |  | 1 | |  |  |  | |  |  | | 1 | 1 | 1 | 1 | 1 |  |  | 0 |  | 1 | 1 | 1 | 0 |  | 1 | 0 | |
| YOR161C-C | 37 | 48 | 1 | 1 |  |  | 1 |  |  | 1 | 1 |  |  | 1 | |  | 0 | | 1 |  |  | |  |  |  | |  | 1 | | 1 | 1 |  | 1 | 1 |  |  |  | 1 |  |  |  | 1 |  |  |  | |
| YPL038W-A | 24 | 63 | 0 |  | 0 |  |  |  |  |  | 0 | 0 | 0 |  | |  |  | |  |  |  | |  |  | 1 | |  |  | | 1 | 1 | 0 | 1 |  |  |  |  | 0 | 0 |  |  | 1 | 0 | 0 | 0 | |
| ZPS1 | 38 | 249 | 0 |  |  |  |  |  |  |  |  | 0 | 1 | 0 | |  | 1 | | 1 |  | 1 | |  |  |  | |  | 1 | | 1 | 1 |  | 1 | 1 | 1 |  | 1 |  | 1 |  |  | 0 |  |  |  | |
