# Supplementary material for: Trait Variation in Yeast Is Defined by Population History
Source: PLoS Genet. 2011 Jun 16;7(6):e1002111. doi: 10.1371/journal.pgen.1002111 (PMC3116910; doi:10.1371/journal.pgen.1002111)
Supplement: Table S5 — Copy Number Variations in natural yeast isolates. CNVs in S. cerevisiae with product P-value of less than e-50 (see Text S1). For each gene in each strain, the ‘copy number ratio’ was computed as log[1+observed reads]−log[1+expected reads]. Dubious genes and transposable elements, which represented the most highly variable genes, were excluded. (DOC) [file pgen.1002111.s024.doc]

**Table S5 Copy Number Variations in natural yeast isolates**

CNVs in *S. cerevisiae* with product P-value of less than e-50 (see Supplementary Materials and Methods). For each gene in each strain, the ‘copy number ratio’ was computed as log[ 1 + observed reads ] – log[ 1 + expected reads]. Dubious genes and transposable elements, which represented the most highly variable genes, were excluded.

|  | European | | | | | | | | | | | | NA | | Malaysian | | | Sake | | | WA | | Mosaics | | | | | | | | | | | | | | | | |
| --- | --- | --- | --- | --- | --- | --- | --- | --- | --- | --- | --- | --- | --- | --- | --- | --- | --- | --- | --- | --- | --- | --- | --- | --- | --- | --- | --- | --- | --- | --- | --- | --- | --- | --- | --- | --- | --- | --- | --- |
| Gene | RM11_1A | L_1374 | L_1528 | BC187 | YJM975 | YJM978 | YJM981 | DBVPG1106 | DBVPG1373 | DBVPG1788 | DBVPG1853 | DBVPG6765 | YPS128 | YPS606 | UWOPS03_461_4 | UWOPS05_217_3 | UWOPS05_227_2 | Y12 | Y9 | K11 | DBVPG6044 | NCYC110 | SK1 | Y55 | 273614N | 322134S | 378604X | DBVPG6040 | LEO | NCYC361 | S288c | UWOPS83_787_3 | UWOPS87_2421 | W303 | YIIc17_E5 | YJM789 | YS2 | YS4 | YS9 |
| YLR162W | -2.9 | -1.8 | -1.8 | -1.4 | -1.8 | -1.8 | 2.6 | -1.3 | -2 | -1.8 | 1.9 | -2.9 | 1.6 | 1.3 | -1.7 | -1.7 | -1.7 | -1.6 | -1.6 | -1.6 | -2.2 | -1.5 | -3 | -3 | -1.6 | -1.6 | -1.7 | -1.5 | -0.7 | -1.2 | -2 | -1.6 | -1.7 | -2.5 | -1.7 | -2.9 | 1 | 1.6 | 1 |
| YLR154C-G | -2.9 | -1.8 | -1.8 | -1.4 | 0.9 | 1 | 2.4 | -1.3 | -2 | -1.8 | -1.7 | -2.9 | 1.1 | 1.2 | -1.7 | -1.7 | -1.7 | -1.6 | 1.2 | -1.6 | -2.2 | -1.5 | -3 | -0.3 | -1.6 | -1.6 | -1.7 | -1.5 | -0.7 | -1.2 | -2 | -1.6 | -1.7 | -2.5 | 1.4 | -2.9 | 1.4 | 1.3 | 1 |
| RRT15 | -1.6 | -1.8 | -1.8 | -1.4 | 1.5 | 1.7 | 2.5 | -1.3 | -2 | -1.8 | -1.7 | -2.9 | 0.9 | 1.2 | -1 | -1 | -1.7 | -1.6 | -1.6 | -1.6 | -1.5 | -1.5 | -2.3 | -1.6 | -1.6 | -1.6 | -1.7 | -0.4 | -0.7 | -1.2 | -2 | -1.6 | -1 | -1.8 | 0.6 | -2.9 | 1.2 | 1.4 | 0.8 |
| YIL080W | -2.9 | -1.8 | -1.8 | -1.4 | -1.8 | -1.8 | -1.9 | -1.3 | -0.2 | -1.8 | -1.7 | -2.9 | 1.7 | 1.4 | -1.7 | -1.7 | -1.7 | 0.5 | 0.5 | 0.1 | -0.1 | 0.1 | -0.5 | 0.1 | 1.2 | -1 | 0.5 | 0.9 | -0.7 | -0.1 | 0.1 | 1 | 1 | 0.9 | -0.1 | -2.9 | 0 | -0.4 | -1.7 |
| NUM1 | 1.6 | -0.9 | -1.2 | 0 | -0.3 | -0.6 | -1.5 | -0.6 | 0.7 | 0.1 | -1.4 | -1.6 | -0.3 | -0.2 | -0.4 | -1.3 | 0.2 | -0.5 | 0.5 | -1 | -0.5 | -2.2 | -0.8 | -1.5 | 1.2 | -1.7 | -1.4 | -0.4 | -0.5 | -1.2 | 0 | -1.3 | -0.2 | 0.2 | -1.8 | 0.5 | -1.4 | -0.8 | -0.3 |
| YRF1-6 | 0.7 | 0 | -0.8 | -1.4 | -1.8 | 0 | 2.5 | -1.4 | -0.3 | -1.2 | -1.1 | -2.2 | -1.4 | -0.8 | -1.7 | -1.7 | -1.8 | -1.6 | -0.9 | -1 | -2.2 | -1.6 | -2.3 | -3 | -1 | -0.6 | -0.7 | -1.6 | -0.7 | -1.3 | -1.3 | -1.7 | -1.7 | 0.1 | -0.7 | 1.5 | -0.7 | -0.5 | -0.7 |
| YRF1-7 | 0.7 | 0 | -0.8 | -1.4 | -1.8 | -0.2 | 2.5 | -1.4 | -0.3 | -1.2 | -1.1 | -2.2 | -1.4 | -0.8 | -1.7 | -1.7 | -1.8 | -1.6 | -0.9 | -1 | -2.2 | -1.6 | -2.3 | -3 | -1 | -0.6 | -0.7 | -1.6 | -0.7 | -1.3 | -0.9 | -1.7 | -1.7 | 0 | -0.7 | 1.5 | -0.7 | -0.8 | -0.7 |
| YRF1-3 | 0.7 | 0 | -0.8 | -1.4 | -1.8 | -0.2 | 2.5 | -1.4 | -0.3 | -1.2 | -1.1 | -2.2 | -1.4 | -0.8 | -1.7 | -1.7 | -1.8 | -1.6 | -0.9 | -1 | -2.2 | -1.6 | -2.3 | -3 | -1 | -0.6 | -0.7 | -1.6 | -0.7 | -1.3 | -0.9 | -1.7 | -1.7 | 0 | -0.7 | 1.5 | -0.7 | -0.8 | -0.7 |
| YRF1-5 | 0.7 | -0.2 | -0.5 | -1.4 | -1.8 | -0.2 | 2.5 | -0.7 | -0.5 | -0.5 | -1.1 | -2.2 | -1.4 | -0.8 | -1.7 | -1 | -1.8 | -0.5 | -0.9 | -1.7 | -2.2 | -1.6 | -2.3 | -3 | -1.7 | -0.1 | -0.7 | -1.6 | -0.7 | -1.3 | -1.3 | -1.7 | -1.7 | 0.3 | -0.7 | 1.4 | -0.3 | -0.2 | -0.4 |
| YRF1-8 | 0.7 | -0.2 | -0.5 | -1.4 | -1.8 | -0.2 | 2.5 | -0.7 | -0.5 | -0.5 | -1.1 | -2.2 | -1.4 | -0.8 | -1.7 | -1 | -1.8 | -0.5 | -0.9 | -1.7 | -2.2 | -1.6 | -2.3 | -3 | -1.7 | -0.1 | -0.7 | -1.6 | -0.7 | -1.3 | -1.3 | -1.7 | -1.7 | 0.3 | -0.7 | 1.4 | -0.3 | -0.2 | -0.4 |
| YRF1-1 | 0.7 | -0.2 | -0.5 | -1.4 | -1.8 | -0.2 | 2.5 | -0.7 | -0.5 | -0.5 | -1.1 | -2.2 | -1.4 | -0.8 | -1.7 | -1 | -1.8 | -0.5 | -0.9 | -1.7 | -2.2 | -1.6 | -2.3 | -3 | -1.7 | -0.1 | -0.7 | -1.6 | -0.7 | -1.3 | -1.3 | -1.7 | -1.7 | 0.3 | -0.7 | 1.4 | -0.3 | -0.2 | -0.4 |
| YLL066C | 0.7 | -0.2 | -0.8 | -1.4 | -1.8 | 0.3 | 2.8 | -1.4 | -0.9 | -1.9 | -1.7 | -1.3 | -2.1 | -2.2 | -1.7 | -1.7 | -1.8 | -0.5 | -1.6 | -1.7 | -2.2 | -1.5 | -3 | -3 | -0.9 | 0.1 | -0.7 | -1.6 | -0.7 | -1.3 | -0.2 | -1.7 | -1.7 | 0.5 | -0.7 | 1 | -0.3 | -0.5 | -0.2 |
| YRF1-2 | 0.4 | 0 | -0.8 | -1.4 | -1.8 | 0.1 | 2.8 | -1.4 | -0.7 | -1.2 | -1.8 | -1.8 | -1.4 | -0.6 | -1.7 | -1 | -1.8 | -1.6 | -0.9 | -1.7 | -2.2 | -1.5 | -1.9 | -3 | -1.6 | -0.6 | -0.7 | -1.6 | -0.7 | -1.3 | -1.3 | -1.7 | -1.7 | 0.1 | -0.7 | 1.3 | -0.3 | -0.5 | -0.2 |
| YLL067C | 0.6 | -0.4 | -0.8 | -1.4 | -1.8 | -0.2 | 2.7 | -1.4 | -1.4 | -1.9 | -1.7 | -1.1 | -2.1 | -2.2 | -1.7 | -1 | -1.1 | -0.5 | -1.6 | -1.7 | -1.5 | -1.5 | -1.9 | -3 | -0.5 | 0.4 | -1.1 | -1.6 | -0.7 | -1.3 | -0.4 | -1.7 | -1 | 0.3 | -1.1 | 1.1 | 0 | -0.5 | 0 |
| YRF1-4 | 0.4 | -0.2 | -0.8 | -1.4 | -1.8 | 0 | 2.8 | -1.4 | -0.9 | -1.9 | -1.7 | -1.5 | -1.4 | -0.8 | -1.7 | -1.7 | -1.1 | -0.5 | -0.9 | -1.7 | -2.2 | -1.5 | -2.3 | -3 | -1.6 | -0.1 | -0.7 | -0.9 | -0.7 | -1.3 | -0.2 | -1.7 | -1.7 | 0.3 | -0.7 | 1.1 | -0.3 | -0.2 | -0.2 |
| YPR204W | 0.1 | -0.7 | -1.9 | -1.4 | -1.1 | 0 | 2.9 | -1.4 | -1 | -1.9 | -1.8 | -1 | -2.1 | -1.5 | -1.7 | -1 | -1.1 | -0.2 | -0.5 | -1.7 | -2.2 | -1.5 | -1.4 | -3 | -1.6 | 0.5 | -0.7 | -1.6 | -0.7 | -1.3 | -0.6 | -1 | -1 | 0.2 | -1.1 | 0.9 | 0.6 | -0.7 | 0 |
| YIL177C | 0.4 | -0.7 | -0.8 | -1.4 | -1.8 | -1.1 | 2.1 | -1.4 | -1 | -0.8 | -1.1 | -2.2 | -0.7 | 0.4 | -1.7 | -1 | -1.8 | 0 | 0 | -0.3 | -1.5 | -1.5 | -0.4 | -2.3 | -1.6 | -0.6 | -0.7 | 0 | -0.7 | -1.3 | -0.9 | -1 | -1.7 | -0.1 | -1.1 | 1.4 | 0.2 | 0.1 | 0.4 |
| TAR1 | 1.2 | 0.6 | -0.7 | -1.4 | -0.2 | -0.7 | 1.2 | -1.3 | -2 | -1.8 | 0.6 | 0.1 | -0.4 | 0.1 | 0.4 | 0.6 | 0.3 | 0.2 | 0.4 | -0.5 | -2.2 | -0.8 | -3 | -3 | -1.6 | -1.6 | -1.7 | 0.6 | -0.7 | 0.6 | 0.6 | -0.5 | -0.3 | 0.8 | 0.1 | -2.9 | -1.4 | 0.4 | 0.1 |
| YJL225C | 0.4 | -0.7 | -0.8 | -1.4 | -1.8 | -1.1 | 2.1 | -1.4 | -1 | -0.8 | -1.1 | -2.2 | -0.7 | 0.4 | -1.7 | -1 | -1.8 | -0.2 | 0.2 | -0.3 | -1.5 | -1.5 | -0.3 | -2.3 | -1.6 | -0.6 | -0.7 | 0 | -0.7 | -1.3 | -0.9 | -1 | -1.7 | -0.1 | -1.1 | 1.4 | 0.2 | 0.1 | 0.4 |
| YHR219W | -1 | 0.3 | -1.2 | -0.7 | -1.8 | 1 | 2.9 | -1.3 | -0.9 | -1.9 | -1.7 | -2.9 | -1.4 | -1.5 | -1.7 | -1 | -1.1 | 0 | 0.4 | -1.7 | -2.2 | -0.8 | -0.9 | -2.3 | -0.9 | 0.9 | -0.7 | -1.5 | -0.7 | -1.2 | 0.2 | -1.7 | -1.7 | 0.4 | -1.1 | 0.3 | 0.2 | 0 | -0.4 |
| YBL113C | -1 | 0.1 | -1.2 | -0.7 | -1.8 | 0.8 | 2.9 | -1.4 | -0.9 | -1.9 | -1.1 | -2.9 | -1.4 | -1.5 | -1.7 | -0.6 | -1.1 | 0.2 | 0.5 | -1.7 | -2.2 | -0.8 | -0.5 | -2.3 | -0.9 | 0.9 | -1.1 | -1.6 | -0.7 | -1.2 | 0.1 | -1.7 | -1.7 | 0.4 | -1.8 | 0.3 | 0.2 | 0.1 | -0.4 |
| YEL077C | 0.1 | -1.1 | -0.7 | -1.4 | -1.8 | -0.7 | 2.7 | -1.3 | -1.3 | -1.9 | -1.7 | -1.5 | -1 | -0.8 | -1.7 | -1 | -1.1 | -0.2 | 0.4 | -1.7 | -1.5 | -0.8 | -0.3 | -3 | -1.6 | 0.7 | -1.7 | -0.8 | -0.7 | -1.2 | -0.9 | -1 | -1 | 0 | -1.1 | 1 | 0.7 | 0 | 0.2 |
| YHL050C | -0.1 | 0 | -1.1 | -0.7 | -1.8 | 0.6 | 2.8 | -1.3 | -0.9 | -1.1 | -1.7 | -2.2 | -1.4 | -1.5 | -1.7 | -1 | -1 | -0.2 | 0.4 | -1.6 | -2.2 | -1.5 | -0.8 | -2.3 | -1.6 | 1.1 | -1 | -0.8 | -0.7 | -1.2 | -0.4 | -1.6 | -1 | 0.3 | -1 | 0.6 | 0.6 | 0 | 0.2 |
| YFL068W | -1.5 | -0.2 | -1.1 | -1.4 | -1.1 | 1.2 | 2.9 | -1.3 | -1.3 | -1.8 | -1.7 | -1.8 | -1.3 | -2.2 | -1.6 | -0.6 | -0.6 | 0.2 | 0.1 | -0.9 | -2.2 | -0.4 | -0.5 | -1.6 | -0.5 | 1.1 | -1 | -0.8 | -0.7 | -0.5 | -0.2 | -0.5 | -1 | 0.2 | -1 | -0.3 | -0.2 | 0 | -0.6 |
| YLL067W-A | -1.5 | -0.2 | -1.1 | -1.4 | -1.1 | 1.2 | 2.9 | -1.3 | -1.3 | -1.8 | -1.7 | -1.8 | -1.3 | -2.2 | -1.6 | -0.6 | -0.6 | 0.2 | 0.1 | -0.9 | -2.2 | -0.4 | -0.5 | -1.6 | -0.5 | 1.1 | -1 | -0.8 | -0.7 | -0.5 | -0.2 | -0.5 | -1 | 0.2 | -1 | -0.3 | -0.2 | 0 | -0.6 |
| YEL077W-A | -1.5 | -0.2 | -1.1 | -1.4 | -1.1 | 1.2 | 2.9 | -1.3 | -1.3 | -1.1 | -1.7 | -1.8 | -1.3 | -2.2 | -1.6 | -0.6 | -0.6 | 0.2 | 0.1 | -0.9 | -2.2 | -0.4 | -0.4 | -1.6 | -0.5 | 1.1 | -1 | -0.8 | -0.7 | -0.5 | 0 | -0.5 | -1 | 0.2 | -1.7 | -0.1 | -0.3 | 0 | -0.6 |
| YPR158W-B | -0.1 | -1.8 | -1.8 | -1.4 | -1.8 | -1.8 | -1.9 | -1.3 | -0.9 | -1.8 | -0.1 | -2.9 | -2 | -2.2 | -1.7 | -1.7 | -1.7 | -0.5 | -0.2 | -1 | 0.4 | -0.8 | 0.8 | -0.8 | 0.2 | 0 | -0.3 | -0.1 | -0.7 | -1.2 | -0.6 | -1.6 | 0.3 | 0.7 | -1 | 1.8 | -1.4 | -1.1 | -0.1 |
| YHL050W-A | -1.5 | -0.2 | -1.1 | -1.4 | -1.1 | 1.2 | 2.9 | -1.3 | -1.3 | -1.8 | -1.7 | -1.8 | -1.3 | -2.2 | -1.6 | -0.6 | -0.6 | 0.2 | 0.1 | -0.9 | -2.2 | -0.4 | -0.3 | -1.6 | -0.5 | 1.1 | -1 | -0.8 | -0.7 | -0.5 | -0.2 | -0.5 | -1 | 0.2 | -1 | -0.3 | -0.2 | 0 | -0.6 |
| YDR316W-B | -0.2 | -1.8 | -1.8 | -1.4 | -1.8 | -1.8 | -1.9 | -1.3 | -0.9 | -1.8 | 0.1 | -2.9 | -2 | -2.2 | -1.7 | -1.7 | -1.7 | -0.9 | -0.2 | -1 | 0.4 | -0.8 | 0.8 | -0.9 | -0.2 | 0.1 | -0.3 | -0.1 | -0.7 | -1.2 | -0.4 | -1.6 | 0.1 | 0.7 | -1 | 1.8 | -1.4 | -1.1 | -0.1 |
| YDR098C-B | 0.9 | -1.8 | -1.1 | -1.4 | -1.8 | -1.8 | -1.9 | -1.3 | -0.9 | -1.1 | -1.7 | -1.5 | -2 | -2.2 | -1.6 | -1.7 | -1.7 | -0.5 | -0.5 | -0.9 | -0.1 | -0.8 | 0.4 | -1.2 | -0.9 | -0.5 | -0.3 | -0.8 | -0.7 | -1.2 | -1.3 | -1.6 | 0.3 | 0.2 | -0.6 | 1.8 | -0.7 | -1.1 | -0.1 |
| YER138C | -0.3 | -1.8 | -1.8 | -1.4 | -1.8 | -1.8 | -1.9 | -1.3 | -2 | -1.8 | 0.1 | -2.9 | -2 | -2.2 | -1.7 | -1.7 | -1.7 | -0.2 | -0.9 | -0.5 | 0.3 | -0.8 | 0.4 | -0.6 | -0.2 | 0.1 | -0.6 | -0.1 | -0.7 | -1.2 | 0.6 | -1.6 | -0.3 | 0.9 | -1 | 1.8 | -1.4 | -0.7 | -0.4 |
| YAR009C | 0.6 | -1.8 | -1.8 | -1.4 | -1.8 | -1.8 | -1.9 | -1.3 | -2 | -1.8 | 0.5 | -2.9 | -2 | -1.1 | -1.7 | -1.7 | -1.7 | -0.5 | -0.2 | -1.6 | 0.1 | -0.4 | 0.7 | -0.5 | -0.2 | 0.3 | -0.6 | -0.8 | -0.7 | -1.2 | 0.7 | -0.9 | 0.1 | 0.5 | -1 | 1.5 | -1.4 | 0 | -0.4 |
| YPR158C-D | -0.1 | -1.8 | -1.8 | -1.4 | -1.8 | -1.8 | -1.9 | -1.3 | -2 | -1.8 | 0.1 | -2.9 | -2 | -2.2 | -1.7 | -1.7 | -1.7 | -0.9 | -0.5 | -1 | 0.5 | -0.4 | 0.6 | -0.4 | 0 | 0.1 | -0.6 | -0.1 | -0.7 | -1.2 | 0.3 | -1.6 | -0.3 | 0.7 | -1 | 1.8 | -1.4 | -1.8 | -0.6 |
| YER190C-B | -1.5 | -0.3 | -1.1 | -1.3 | -1 | 1.2 | 2.9 | -1.3 | -1.2 | -1.8 | -1.6 | -1.7 | -1.3 | -2.1 | -1.6 | -0.5 | -0.6 | 0.1 | 0.1 | -0.9 | -2.1 | -0.3 | -0.5 | -1.5 | -0.4 | 1.1 | -1 | -0.8 | -0.6 | -0.5 | -0.1 | -0.9 | -0.9 | 0.3 | -1.7 | -0.1 | -0.6 | 0.1 | -0.6 |
| YHR054C | -1.2 | -1.6 | 1 | -0.6 | 0.6 | -0.9 | -1.7 | -0.1 | 1.5 | 0.1 | -0.9 | -0.1 | -0.3 | -0.6 | -0.8 | -0.4 | 0 | 0.2 | -0.7 | -0.4 | -0.6 | 0.6 | -1.2 | -0.1 | -0.8 | 0.4 | 0 | -0.7 | -0.6 | -0.4 | 1.4 | -1.5 | -0.4 | 1.3 | -0.9 | -1.1 | -0.5 | -1 | -0.9 |
| YHR218W | 0.4 | -1.1 | -0.2 | -0.7 | -0.7 | -0.4 | 2.7 | -1.3 | -1.3 | -1.8 | -1 | -1.1 | -2 | -1.5 | -1.7 | -1.7 | -1 | -0.9 | -0.5 | -1.6 | -1.1 | -1.5 | -0.6 | -2.3 | -0.2 | 0.8 | -0.6 | -0.8 | -0.7 | -0.1 | -0.4 | -1.6 | -0.3 | 0.3 | -1 | 0.5 | 0 | 0.1 | -0.1 |
| YEL076C-A | -2.3 | -0.7 | -1.2 | -0.3 | -1.1 | 0.4 | 2.7 | -0.7 | -0.4 | -0.5 | -0.4 | -2.2 | 0 | -0.1 | -1 | -0.1 | -0.7 | 0 | 0.2 | -1 | -2.2 | -1.5 | -0.3 | -1.6 | -1.6 | 1.4 | -0.4 | 0.1 | -0.7 | -1.2 | -0.1 | -1.6 | -0.3 | -0.2 | -0.1 | 0.2 | 0.2 | -0.2 | -0.4 |
| YEL076C | -2.2 | -0.3 | -1.1 | -0.2 | -1 | 0.6 | 2.8 | -0.6 | -0.3 | -0.7 | -1 | -2.1 | -0.4 | -0.1 | -0.9 | 0.2 | -1 | -0.4 | 0.3 | -0.9 | -2.1 | -1.4 | -0.8 | -2.2 | -0.8 | 1.4 | -0.6 | 0.5 | -0.6 | -0.5 | -0.3 | -1.6 | -0.5 | -0.2 | -0.6 | 0.1 | 0.1 | -0.1 | -0.3 |
| YBL111C | 0.5 | -0.7 | 0 | -1.4 | -1.1 | -0.2 | 2.7 | -1.3 | -0.6 | -1.1 | -1 | -0.9 | -1.4 | -1.1 | -1.7 | -1.7 | -1.7 | -1.6 | -0.2 | -0.3 | -1.5 | -1.5 | -0.7 | -2.3 | -0.2 | 0.5 | -0.6 | -0.8 | -0.7 | -0.5 | 0 | -1.6 | -1 | 0.3 | -0.6 | 0.6 | 0.4 | -0.2 | -0.4 |
| YLR464W | -1.9 | -0.7 | -1.2 | -0.3 | -1.1 | 0.3 | 2.7 | -0.6 | -0.4 | -0.5 | -0.3 | -2.2 | 0.1 | -0.1 | -1 | -0.1 | -0.7 | -0.2 | 0.2 | -1.7 | -2.2 | -1.5 | -0.3 | -1.6 | -1.6 | 1.4 | -0.4 | 0.1 | -0.7 | -1.2 | -0.1 | -1.6 | -0.3 | -0.3 | -0.1 | 0.1 | 0.2 | 0 | -0.4 |
| YFL066C | -0.4 | -0.7 | -1.1 | -1.4 | -0.4 | -0.4 | 2.9 | -1.3 | -1.3 | -1.8 | -0.6 | -1.1 | -2 | -1.5 | -1.7 | -1.7 | -1 | -0.5 | 0 | -1.6 | -1.1 | -0.4 | -0.5 | -1 | -1.6 | 1.2 | -0.6 | -0.8 | -0.7 | -0.5 | -0.2 | -1.6 | -0.6 | 0.1 | -1 | 0 | 0.8 | 0 | -0.1 |
| YER138W-A | 0.2 | -0.2 | 0.7 | 0 | 0.5 | -0.4 | -0.8 | 0.3 | -0.4 | 0.5 | -0.3 | 1.1 | -2 | -2.2 | -1.6 | -1.7 | -1.7 | -1.6 | -1.6 | -0.9 | -2.2 | -1.5 | -0.7 | -0.5 | 0 | 0 | -1 | -0.4 | -0.7 | 0.9 | 0 | -0.9 | -0.6 | -0.3 | 0.4 | 0.6 | 0.6 | 0.6 | 0.5 |
| YLR462W | -0.5 | -0.4 | -1.1 | -1.4 | -0.7 | 0.8 | 2.6 | -1.3 | -0.2 | -0.5 | -1 | -0.7 | -1 | -0.1 | -1.7 | -1 | -1.7 | 0 | 0 | 0.1 | -2.2 | -1.5 | -0.8 | -1.6 | -0.5 | 0.4 | -0.6 | -0.8 | -0.7 | -0.1 | 0.4 | -0.2 | -0.6 | 0.3 | -0.6 | 0 | 0.2 | 0.4 | -0.4 |
| YFL064C | -0.5 | -0.2 | -1.1 | -1.4 | -0.7 | 0.9 | 2.7 | -1.3 | 0.1 | -0.5 | -0.6 | -0.7 | -1.4 | -0.1 | -1.7 | -1 | -1.7 | 0.2 | 0 | 0 | -2.2 | -1.5 | -0.8 | -1.6 | -0.5 | 0.4 | -0.6 | -0.8 | -0.7 | -0.1 | 0 | -0.5 | -0.6 | 0 | -0.6 | 0 | 0.3 | 0.1 | -0.4 |
| YHL049C | -0.5 | -0.7 | -0.7 | -1.4 | -0.2 | 0.6 | 2.7 | -1.3 | -0.4 | -0.5 | -0.6 | -0.8 | -0.7 | -0.2 | -1.7 | -1 | -1.7 | -0.2 | 0 | 0.1 | -1.5 | -1.5 | -0.7 | -1.6 | -0.5 | 0.7 | -1 | -1.5 | -0.7 | -0.1 | 0.1 | -0.5 | -0.6 | 0.3 | -0.6 | -0.1 | 0.3 | 0.1 | -0.6 |
| YOR192C-C | -1.8 | -1.8 | -1.1 | -0.7 | -0.1 | 0.2 | -1.9 | -0.6 | -1.3 | -0.7 | -1 | -1.5 | 1.1 | 0.8 | 0.6 | 0.3 | -1 | -0.2 | -0.8 | -0.5 | 0.7 | 0.6 | 0.4 | 0.5 | 0.4 | -0.2 | -0.6 | -0.8 | -0.7 | -0.1 | 0.2 | 1.1 | -0.3 | -0.4 | -0.6 | -0.1 | 0.1 | 0.5 | -0.1 |
| YPR202W | -0.6 | 0.3 | -1.1 | -0.7 | -0.4 | 0.8 | 2.6 | -0.6 | 0.1 | -0.7 | -0.3 | -0.5 | -2.1 | -0.4 | -1.7 | -1 | -1 | -0.2 | 0.4 | 0 | -1.5 | -1.5 | -0.4 | -1.2 | 0 | 0.1 | -0.3 | -0.4 | -0.7 | -0.5 | 0.1 | -0.2 | -0.6 | -0.1 | -0.6 | 0 | 0.4 | 0.3 | -0.4 |
| ENA2 | -0.7 | 0.6 | -0.6 | -0.2 | 0 | 0 | -0.1 | -0.1 | -0.1 | -0.1 | -0.9 | 0.6 | -0.8 | -0.9 | -0.4 | -1.5 | -0.5 | 0.2 | 0 | -0.8 | -1.3 | -0.3 | -1.4 | -0.7 | -0.4 | -0.1 | 0.8 | 0.7 | 0.1 | -1.1 | 1.4 | -0.8 | -0.1 | 1 | -0.5 | 0 | -0.5 | 0.8 | 0 |
| VTH2 | -1.1 | -1.1 | -1.1 | 0 | -0.4 | -0.7 | -1.9 | -0.6 | -0.4 | -0.7 | -0.3 | -2.9 | 0.4 | 0 | 0 | 0.1 | 0.2 | 0.2 | 1.1 | 0.6 | 0.5 | 0.3 | 0.8 | 0.2 | 0 | -0.5 | -0.6 | -0.4 | -0.7 | -1.2 | 0.2 | 0 | 0.1 | 0.6 | -0.3 | -0.4 | -1.3 | 0.4 | 0.7 |
| YPR203W | -0.5 | -0.6 | -0.6 | -0.2 | -0.5 | 0 | 2.7 | -1.2 | -0.8 | -0.6 | -0.5 | -1.3 | -0.3 | -0.6 | -1.5 | 0.1 | -0.9 | -0.8 | -0.7 | -0.4 | 0.2 | -0.7 | 0.1 | -0.4 | -1.5 | 0.6 | -0.9 | -0.7 | -0.6 | -1.1 | -0.5 | -1.5 | -1.5 | 0.1 | -0.9 | -0.1 | 0.1 | -0.3 | 0.2 |
| VTH1 | -1.1 | -1.1 | -1.1 | 0 | -0.4 | -0.7 | -1.9 | -0.6 | -0.4 | -0.7 | -0.3 | -2.2 | 0.4 | 0 | 0 | 0.1 | 0.2 | 0.2 | 1.1 | 0.6 | 0.5 | 0.3 | 0.8 | 0.2 | 0 | -0.5 | -0.6 | -0.4 | -0.7 | -1.2 | 0.2 | 0 | 0.1 | 0.6 | -0.3 | -0.4 | -1.3 | 0.4 | 0.7 |
| *CUP1*-2 | 0.7 | -1.3 | 0.5 | -0.9 | 0.7 | -0.6 | -0.3 | -0.9 | 1.8 | -0.6 | -0.1 | -0.5 | -1.5 | -0.2 | -1.2 | -1.2 | -1.2 | 0.3 | -0.4 | -1.2 | -0.5 | 0.1 | -0.8 | -0.1 | 0.5 | -0.1 | -0.5 | -0.4 | -0.4 | -0.8 | 0.3 | -0.4 | -1.2 | 0.9 | -0.5 | -0.7 | 0.2 | -0.6 | -0.1 |
| *CUP1*-1 | 0.7 | -1.3 | 0.5 | -0.9 | 0.7 | -0.6 | -0.3 | -0.9 | 1.8 | -0.6 | -0.1 | -0.5 | -1.5 | -0.2 | -1.2 | -1.2 | -1.2 | 0.3 | -0.4 | -1.2 | -0.5 | 0.1 | -0.8 | -0.1 | 0.5 | -0.1 | -0.5 | -0.4 | -0.4 | -0.8 | 0.3 | -0.4 | -1.2 | 0.9 | -0.5 | -0.7 | 0.2 | -0.6 | -0.1 |
| FLO5 | 0.3 | 0.6 | 0 | -0.3 | -1.3 | 0.6 | -1.5 | 0.1 | 1.5 | 1.1 | -0.6 | -0.4 | 0.2 | -0.6 | -0.6 | -0.2 | -0.6 | -1.2 | -1.2 | -1.2 | -0.6 | -0.4 | -1.1 | -1.1 | -0.1 | -0.6 | -1.3 | -1.1 | -0.4 | 0.2 | -0.4 | 0.2 | -0.2 | 0.5 | 1 | 0.6 | -1 | -0.7 | 0.1 |
| HKR1 | 0.4 | 0.2 | -0.9 | -1.6 | 0.5 | -0.9 | -1.4 | 0.7 | -2.2 | -0.6 | 0.3 | -0.2 | 0.1 | -0.6 | -0.7 | -1.1 | 0.2 | 0.2 | -0.1 | -0.7 | -1 | -0.1 | 0 | -0.1 | 0.5 | 0 | 0.7 | -0.1 | -0.8 | -0.3 | -0.2 | 0.7 | 0.8 | 0.1 | -0.3 | 0.4 | -0.4 | 0.7 | -1.9 |
| ENA1 | -0.4 | 0.5 | -0.6 | -0.2 | 0 | 0 | -0.1 | 0.2 | 0 | -0.1 | -0.9 | 0.5 | -0.8 | -0.6 | -0.4 | -1.5 | -0.2 | 0.2 | 0.2 | -0.8 | -1.3 | -0.7 | -1 | -0.6 | -0.8 | 0.1 | 0.8 | 0.7 | 0.1 | -1.1 | 1.1 | -0.8 | -0.1 | 1 | -0.5 | 0 | -0.5 | 0.8 | -0.2 |
| YFL067W | -0.7 | 0.2 | -0.9 | -0.6 | -0.9 | -0.2 | 2.6 | -0.6 | -1.1 | -0.9 | -0.1 | -1.8 | -1.1 | -1.2 | -0.8 | -0.8 | -0.9 | 0 | 0 | -0.8 | -1.2 | -0.7 | 0.5 | -1.1 | -0.1 | 0.6 | 0.3 | 0 | -0.2 | -0.5 | -0.3 | -0.1 | -0.1 | -0.7 | -0.8 | 0.3 | -0.6 | -0.9 | -0.2 |
| MEC1 | -0.2 | 1.1 | 0.9 | 0.5 | 0.6 | -0.2 | -1.2 | 0.1 | -0.1 | 0.8 | 0.4 | 0 | 0.4 | -0.2 | -1 | -0.1 | 0.5 | -0.2 | 0.4 | -0.5 | 0 | -0.4 | -1.4 | -0.2 | -0.5 | 0.3 | 0.3 | -1.5 | -0.7 | 0.4 | 0.2 | -0.9 | 0.3 | 0.1 | -1 | -0.1 | -0.3 | -0.2 | -1 |
| FLO10 | -2.4 | -0.7 | 0 | -0.3 | 0 | 0.2 | -0.8 | 0.8 | 0.6 | 0.5 | -1.3 | -2.4 | -0.5 | 0.2 | -0.1 | -1.3 | 0.8 | 0.2 | 0.2 | 0.5 | 0.1 | 0.7 | 0.2 | 0 | -0.5 | -1.2 | 0.3 | -0.4 | -0.4 | -0.9 | 0.4 | 0.6 | -0.2 | 0.6 | 0.1 | 0.5 | -1 | -1.4 | -1.3 |
| NTE1 | -0.4 | 0.3 | 0.4 | 0.7 | -0.6 | -1 | 0 | -0.2 | -0.6 | -0.4 | 1 | 0.1 | -0.4 | -0.3 | -0.9 | 0 | 0.5 | -0.8 | -0.8 | 0.5 | -0.5 | 0.7 | -0.3 | 0.6 | 0.2 | 0 | 0.6 | -0.8 | -0.6 | -0.1 | 0.1 | 0.4 | 0 | -1.1 | -0.3 | -0.4 | 0.9 | -0.7 | -0.3 |
| IMD2 | -0.9 | -1.5 | 0 | -0.5 | -0.4 | 0.7 | -0.5 | 0 | -0.2 | -0.2 | 0.1 | -0.8 | -1.1 | -1.2 | -0.3 | -0.7 | -0.4 | 0.3 | -0.6 | 0 | 0.7 | 0.7 | 1 | 0.5 | -0.7 | 0.4 | -0.1 | -0.2 | -0.5 | 0.1 | -0.4 | -0.7 | -0.3 | -0.4 | -0.4 | -0.1 | 0.2 | 0.4 | 0.1 |
| ULS1 | -0.2 | -0.9 | -0.9 | 0.2 | 0.1 | -0.2 | -1.7 | 0.5 | 0.2 | 0.5 | 0.6 | -0.3 | 0.1 | 0.8 | 0.4 | -0.8 | -0.4 | -0.7 | 0.6 | 0 | -1.9 | -0.2 | 0.1 | -0.6 | 0.9 | 0.2 | 1.2 | -0.2 | -0.5 | -1 | 0 | -1.4 | 0.5 | -0.2 | -0.8 | -0.2 | 0.6 | 0.5 | -0.4 |
| RDS1 | 0.6 | 0.5 | -0.4 | 0 | -1.4 | 0.5 | -0.4 | 0.4 | -0.2 | 0.6 | -0.2 | 0 | -0.3 | -1.1 | 0.3 | 0.1 | 0.2 | -1.2 | -1.2 | -1.3 | 0.7 | -0.1 | 0.6 | -2.5 | 0.1 | -0.6 | 0.3 | 0.4 | 0.2 | -0.9 | -1.6 | -0.6 | 0.1 | 0.4 | 0.4 | -0.4 | -1 | -1.4 | -0.7 |
| CSF1 | 0.1 | -0.7 | 0.1 | 0.7 | -0.2 | -0.4 | -1.9 | -1.3 | 0.5 | 0.4 | 0.9 | -0.3 | 0 | -0.2 | -1 | -1 | -0.6 | -0.9 | -0.9 | -0.5 | 0.2 | -0.1 | 0 | 0.3 | 0.2 | 0 | 0.2 | -1.5 | 0.4 | -1.2 | 0 | 1.1 | 0.1 | -0.4 | 0.2 | 0 | 0.3 | -0.2 | 0.1 |
| NFT1 | -2.4 | 0.6 | 0 | -1 | 0.5 | -0.2 | -0.7 | 0.2 | 0.4 | -0.3 | -1.2 | -2.3 | -0.2 | 0.4 | 0.2 | 0.2 | 0.1 | 0.7 | 0 | -0.5 | 0.1 | 0.7 | 0.2 | 0.5 | -1.2 | -1.2 | 0.5 | 0.3 | -0.4 | 0.3 | -0.4 | 1 | 0.4 | 0.1 | 0.1 | -0.2 | -0.9 | -1.3 | -1.3 |
| MUC1 | 1 | 0.7 | 0.2 | 0.1 | -0.2 | -1.4 | -0.8 | 0.7 | 1 | -0.7 | -1.3 | -0.1 | -0.9 | 0.2 | -0.5 | 0.1 | -0.2 | -0.1 | -0.5 | -0.1 | 0.1 | -1.1 | -0.3 | -0.8 | 0.2 | -1.2 | 0.3 | 0 | -0.4 | -0.9 | 0.1 | -0.1 | -1.3 | -0.4 | -1.3 | 0 | 0.1 | -0.7 | 0.5 |
| CIC1 | 1.3 | 0 | 0.4 | 0.6 | 0 | -0.4 | -0.5 | -0.8 | 0.5 | 0.6 | 0.9 | 0 | -1.4 | 0.5 | -0.3 | -0.4 | -1.1 | 0.1 | -0.3 | -1 | -0.1 | -0.2 | -0.6 | -0.6 | 0.1 | -1 | -1.1 | -0.9 | -0.3 | -0.7 | 0.1 | -0.3 | 0.3 | -1.1 | 0.3 | -0.5 | -0.8 | -1.2 | -0.4 |
| APC1 | -0.3 | 0.7 | 0.7 | 0 | 0.5 | -0.4 | -1.2 | 0.5 | 0.5 | 0.6 | 0.6 | -0.4 | -0.6 | 0.4 | 0 | -0.9 | -0.6 | -1.5 | 0.1 | 0 | 0.2 | 0.5 | 0.3 | -0.7 | 0 | -0.5 | -0.1 | 0.7 | 0 | -1.2 | -0.3 | -0.5 | 0.4 | -0.3 | 0.3 | -0.2 | 0.1 | -0.4 | 0.2 |
| COS1 | 0.3 | -0.5 | -0.9 | -0.1 | 0 | -0.2 | -0.1 | -0.5 | -0.7 | -0.5 | -0.4 | 0.5 | 0.1 | -2 | 0.6 | 0.3 | -0.9 | -1.4 | -0.3 | -0.8 | -0.2 | -0.6 | -0.6 | -0.4 | -0.7 | 0.6 | 0.4 | 0.6 | -0.6 | 0.3 | -0.4 | 0.9 | 0.6 | 0.4 | -0.4 | 0.2 | -0.5 | 0.9 | -0.2 |
| YNR065C | -0.8 | -0.4 | -0.4 | 0 | 0.2 | -0.4 | -1.9 | -0.2 | 0.2 | -0.7 | -0.3 | -1.2 | 0.4 | 0.1 | -0.5 | 0 | 0.1 | 0.3 | 0.9 | 0.2 | 0.3 | 0.3 | 0.4 | 0.3 | 0.2 | 0 | -0.6 | -0.4 | -0.6 | -1.2 | 0.1 | 0.4 | 0 | 0.1 | 0.4 | -0.3 | -1.3 | 0.6 | 0.8 |
| CYR1 | -0.1 | -0.4 | 0.1 | -0.7 | 0 | 0 | -1.2 | 0.3 | 0.3 | -0.5 | -1 | -0.5 | -0.3 | 0.5 | 0.3 | -1 | 0.3 | 1 | 0 | 0.8 | 0.2 | -0.8 | 0.4 | 0 | 0.2 | -0.6 | -0.4 | 0.7 | 0.4 | -0.1 | -0.6 | -0.5 | -0.3 | -0.3 | 0.1 | -0.2 | 0.2 | -1.1 | 0.7 |
| FLO1 | 0.4 | -0.1 | 0.2 | -0.9 | -1.2 | -1.2 | -1.3 | -0.8 | 0.2 | 0.7 | -1.1 | -0.6 | 0.8 | 0.4 | 0 | 0 | 0 | -0.3 | -1 | -0.4 | -0.4 | -1 | -0.3 | -1.1 | -1 | -1.1 | -1.1 | -1 | -0.4 | 0.9 | -0.3 | 1 | -0.4 | 0.6 | 0.7 | 0.8 | -0.8 | -0.5 | 0 |
| SLH1 | -0.2 | -1.1 | 0.3 | -1.4 | 0.8 | 0.3 | -1.9 | -0.2 | -0.1 | 0.4 | 0.1 | -0.5 | 0.4 | 0.7 | 0 | -1 | 0.2 | 0 | 0.4 | 0.8 | 0 | 0.1 | 0.1 | 0.1 | -0.2 | 0 | 0.5 | -0.1 | 0 | 0.4 | -0.6 | -0.2 | -0.3 | -0.4 | 0.8 | -0.3 | -1.4 | -0.4 | -1 |
| TRA1 | 0.2 | 0.6 | 0.5 | 0.7 | 0.5 | -0.4 | -1.9 | -0.6 | 0.5 | 0.4 | 0.1 | 0.3 | -0.7 | -0.1 | -0.3 | -1.7 | -0.6 | -0.5 | -0.9 | 0 | -0.4 | 0.3 | -0.3 | 0 | -0.5 | 0.7 | -1.7 | -0.4 | -0.7 | -0.5 | -0.2 | 0.5 | -1.7 | 0.2 | 0.2 | 0.2 | -0.7 | 0 | -0.6 |
| COS6 | 0.1 | -0.4 | -0.8 | -0.4 | 0.2 | -0.4 | -0.9 | -0.4 | -0.6 | -0.8 | -0.7 | 0.4 | 0.5 | -1.8 | 0.7 | 0.3 | -0.7 | -1.3 | -0.2 | -0.6 | -0.2 | 0.2 | -0.2 | -0.3 | -0.2 | 0.6 | 0.2 | -0.5 | -0.5 | 0.1 | -1 | 1.1 | 0.6 | 0.6 | -1.4 | 0.2 | -1.1 | 0.9 | -0.7 |
| PMD1 | -0.3 | -1 | -0.7 | 0.9 | -0.1 | -0.6 | -0.7 | 0.8 | 0.5 | 0.4 | 0.1 | -0.2 | 0.3 | -1.4 | -1.6 | 0.2 | 0.3 | 0.4 | -0.8 | 0 | -1 | -0.7 | 0.1 | 0.3 | -0.4 | -0.9 | 0.1 | -0.1 | -0.6 | -0.5 | 0.2 | 0.7 | 0 | 0 | 0.7 | -0.3 | 0.1 | 0.5 | -0.1 |
| MDN1 | 0.6 | 0.4 | 0.5 | 0 | -0.4 | -0.7 | -0.5 | 0.3 | 0.9 | -0.5 | -1 | 0.2 | -0.1 | 0 | -0.6 | -1 | 0 | -0.2 | -0.9 | -0.3 | -0.2 | 0.1 | 0 | 0.2 | -0.2 | -0.6 | -1 | -0.8 | -0.7 | -0.5 | 0.1 | -1.6 | -0.6 | 0 | -1 | 0.5 | -0.7 | -0.7 | -0.1 |
| SGE1 | 0.5 | -0.5 | -1.2 | -0.9 | -1.1 | -0.1 | -1.2 | 0.3 | 0.6 | 0.4 | 0.3 | 0.6 | 0 | 0.6 | 0.6 | 0 | 0.3 | 0.4 | 0.8 | 0 | 0.1 | -0.9 | -2.2 | -2.2 | 0.6 | -0.4 | 0 | 0.1 | 0.7 | -0.7 | -0.2 | 0.8 | 0 | -0.4 | -1.1 | -0.4 | -0.8 | -0.5 | 0.3 |
| COS2 | 0.3 | -0.4 | -0.9 | -0.1 | 0.3 | -0.1 | 0 | -1.1 | -0.6 | -0.5 | -0.3 | 0.5 | -0.2 | -1.9 | 0.6 | 0.2 | -0.8 | -1.3 | -0.6 | -0.7 | -0.3 | -0.6 | -0.7 | -0.2 | -0.7 | 0.6 | 0.3 | 0.8 | -0.5 | 0.6 | -0.3 | 0.9 | 0.7 | 0.1 | -0.4 | 0.2 | -0.4 | 0.8 | -0.8 |
| BUD4 | -0.4 | 0.1 | 0.7 | 0.5 | 0.5 | 0.9 | -1.1 | -1.2 | -0.3 | -0.1 | 0.7 | -0.1 | 0.3 | 0.2 | 0.2 | -0.9 | 0.1 | 0.3 | 0.3 | 0.4 | 0.1 | -0.3 | 0.5 | -1.1 | 0.3 | -0.5 | -0.3 | -0.3 | 0.5 | -1.1 | -0.3 | 0.2 | -0.2 | -0.3 | -0.9 | -0.4 | 0.7 | -0.1 | -1 |
| COS3 | 0.3 | -0.4 | -0.9 | -0.1 | 0.3 | -0.1 | 0 | -1.1 | -0.6 | -0.5 | -0.4 | 0.5 | -0.2 | -1.9 | 0.5 | 0.2 | -0.8 | -1.3 | -0.6 | -0.7 | -0.1 | -0.6 | -0.6 | -0.2 | -0.7 | 0.5 | 0.3 | 0.8 | -0.5 | 0.6 | -0.3 | 0.9 | 0.7 | 0.1 | -0.4 | 0.2 | -0.4 | 0.8 | -0.8 |
| SNF2 | -0.3 | 0.5 | 0.5 | -0.3 | -1.7 | -0.7 | 0.2 | 0.1 | 0.1 | -0.4 | -0.3 | 0.1 | 0.2 | 0 | 0.5 | 1 | -0.6 | 0.4 | -0.1 | 0.6 | 0.3 | -0.1 | -0.3 | -0.5 | 0.2 | -1.6 | -0.1 | -0.1 | -0.6 | 1 | -0.2 | -0.5 | -0.2 | 0.3 | 0.3 | -0.4 | -1.3 | 0.5 | -0.1 |
| BLM10 | -0.2 | -1.8 | 0.4 | 0.2 | 0 | 0.5 | -1.9 | -1.3 | 0.2 | -0.2 | -0.3 | -0.4 | 0.3 | 0 | -0.3 | -0.3 | -0.3 | -0.9 | -0.2 | 0.3 | -0.6 | -0.8 | 0.3 | 0.5 | -1.6 | 0.1 | -0.1 | 0.1 | -0.7 | -1.2 | 0.4 | 0.9 | 0.5 | 0 | -0.3 | -0.3 | -0.3 | 0.3 | 0.6 |
| NUP157 | -0.3 | 0.3 | -0.1 | -0.2 | 0.2 | -1 | -0.7 | -0.5 | -0.5 | -0.3 | 0.6 | 0 | 0.2 | 0.3 | 0.6 | 0.9 | 0.3 | -0.1 | -0.3 | -0.8 | 0.5 | -0.7 | -0.3 | 0 | -0.1 | -0.1 | -1.6 | 0.7 | -0.6 | 1.1 | -1.2 | 0.7 | -0.1 | -0.1 | 0.5 | -0.5 | 0.1 | 0.3 | -0.5 |
| POL2 | -0.3 | 0 | 0.1 | -0.3 | 0.4 | -0.2 | -1.9 | 0.6 | -0.1 | 0.2 | -0.6 | 0.3 | 0 | 0 | 0.7 | -1 | -1.7 | 0 | -0.2 | 0.8 | -0.4 | -0.1 | -0.1 | -0.5 | -0.9 | 0.1 | 0.7 | 0.4 | 0 | -0.1 | 0 | -0.5 | -0.3 | 0.5 | -0.1 | -0.2 | 0.3 | 0 | 0.6 |
| MAL31 | -0.2 | 0 | -0.3 | 0.1 | -0.9 | -0.2 | -1 | -0.1 | 0.5 | -0.3 | 1 | 0.5 | -0.3 | 0.1 | -0.1 | -0.4 | 0.4 | -0.7 | 0.2 | 0.5 | -1.3 | 0 | -0.3 | 0.2 | 0.2 | 0.8 | 0 | -0.7 | -0.6 | -1.1 | -1.1 | 0.1 | -0.1 | -0.9 | -0.5 | -0.2 | 0.4 | 0.8 | 0.2 |
| FKS1 | -0.2 | 0.3 | 0.6 | 0.7 | -0.1 | -1.8 | -1.9 | -0.6 | -0.9 | -0.7 | -0.3 | 0 | 0.3 | 0 | 0 | 1.3 | 0.2 | -0.9 | -0.2 | -0.2 | 0 | -0.1 | 0.2 | 0.2 | -0.5 | 0 | 0.1 | 0.1 | 0 | 0.2 | -0.6 | 0 | 0 | 0 | 0.1 | -0.4 | 0.4 | -0.4 | 0.4 |
| MYO2 | -0.4 | 0.7 | -1.1 | 0 | 0.6 | -0.4 | -0.5 | 0.3 | -0.1 | 0.1 | -0.3 | 0.2 | -0.2 | 0.4 | -0.9 | 0.5 | -0.1 | -0.2 | 0.6 | -0.2 | -0.4 | -0.1 | 0.3 | 0.3 | -0.2 | 0 | -1 | -0.1 | 0.4 | 0.2 | -2 | -0.9 | 0.3 | -0.4 | 0.6 | -0.5 | 0 | 0.4 | -0.3 |
| SEC7 | -0.2 | -1.1 | -1.1 | 0 | 0 | 0.3 | -1.9 | 0.5 | 0.2 | 0.1 | 0.4 | 0.1 | 0.6 | 0.1 | -1.7 | 0.4 | -0.1 | 0 | 0 | 0.1 | -0.2 | -0.8 | -0.1 | -0.2 | -0.5 | -0.3 | 0.6 | 0.4 | 0 | 1.2 | 0.5 | 0.3 | -0.1 | -0.4 | -0.1 | -0.4 | 0.4 | 0.1 | 0.1 |
