# Supplementary material for: Trait Variation in Yeast Is Defined by Population History
Source: PLoS Genet. 2011 Jun 16;7(6):e1002111. doi: 10.1371/journal.pgen.1002111 (PMC3116910; doi:10.1371/journal.pgen.1002111)
Supplement: Table S6 — Primers used in strain construction. Primers used for inserting the SK1 ENA6 into the URA3 locus of strain BYT5 – a BY4741 carrying a ENA1,2,5 triple deletion. 5′ overhangs correspond to URA3 flanking regions. Gene specific sequences for URA3 or ENA6 at the 3′ end of the primers according to the table. See Text S1 for further details. (DOC) [file pgen.1002111.s025.doc]

**Table S6 Primers used in strain construction**

Primers used for inserting the SK1 *ENA6* into the URA3 locus of strain BYT5 – a BY4741 carrying a *ENA1,2,5* triple deletion. 5’ overhangs correspond to *URA3* flanking regions. Gene specific sequences for *URA3* or *ENA6* at the 3’ end of the primers according to the table. See Supplementary Materials and Methods for further details.

| 5’ overhang forward primers | | **5’AGGTTAATGTGGCTGTGGTTTCGGGTCCATAAAGCTT 3’** | |
| --- | --- | --- | --- |
| 5’ overhang reverse primers | | 5’ TTTTCGTCATTATAGAAATCATTACGACCGAGATTCCC 3’ | |
| Gene | **3’ gene specific sequence forward primer** | | **3’gene specific sequence reverse primer** |
| *URA3* | 5’ AGATTGTACTGAGAGTGCAC 3’ | | 5’ CTGTGCGGTATTTCACACCG 3’ |
| *ENA6* | 5’ ACTTTTGTCAGCCATTCCGTG 3’ | | 5’ TTGGCCATGTTCTTAACCGA 3’ |
