# Supplementary material for: Trait Variation in Yeast Is Defined by Population History
Source: PLoS Genet. 2011 Jun 16;7(6):e1002111. doi: 10.1371/journal.pgen.1002111 (PMC3116910; doi:10.1371/journal.pgen.1002111)
Supplement: Text S1 — Supplementary Materials and Methods. Detailed experimental procedures and protocols. (DOC) [file pgen.1002111.s026.doc]

**SUPPLEMENTARY MATERIALS AND METHODS**

**Strains:** Diploid isolates of *S. cerevisiae*, *S. paradoxus*, *S. arboricolus,* *S. kudriavzevii*, *S. bayanus*, *S. mikatae* and *S. castellii* were collected from diverse sources and geographical locations as described and detailed (Table S1). Strains were long time stored in 20% glycerol at -80C. The universal reference strain BY4741 (MATa; *his3*Δ1; *leu2*Δ0; *met15*Δ0; *ura3*Δ0) , derived from the progenitor S288C, was included as reference. Hybrid strains, for tests of hemizygote growth, were prepared by mating a haploid parental strain to the single deletion strain of interest in the BY4741 background; hybridization was verified by ability to sporulate. The *ENA* triple deletion, BYT5 (*ena1*Δ*2*Δ*5*Δ::loxP, BY4741 derivative), was a kind gift from Hana Sychrová of the Institute of Physiology Academy of Sciences of the Czech Republic. *ENA6* was introduced at the *URA3* locus of the BYT5 mutant in a two-step process. First, *URA3* was reintroduced into its endogenous chromosomal location by PCR amplification from the pRS406 plasmid using 60nt primers (Table S6) followed by transformation into BYT5 with the lithium acetate method . Succesful integration was verified by clone selection after 72h of growth on plates lacking uracil, followed by diagnostic PCR. Clones were selected on *URA* selective medium. Second, the complete *ENA6* region (from 800bp upstream to 300bp downstream) was PCR amplified from genomic DNA extracted from the West African derived SK1 using 60nt primers (Table S6) designed to allow integration into the *URA3* locus and co-transformed with the pRS415 plasmid into the uracil prototrophic BYT5 strain. After 60h of growth on defined leucine drop-out medium at 30°C, transformants were replica plated onto 5-FOA plates. Transformants growing on 5-FOA, were restreaked and replacement of the *URA3* gene with *ENA6* was confirmed by PCR and sequencing. Three independent clones were used for downstream experiments.

**Micro-cultivation:** Strains were subjected to high throughput phenotyping by micro-cultivation (n=2) in an array of environments essentially as previously described . A complete list of environments can be found in Table S2. Strains were inoculated in 350µL of Synthetic Defined (SD) medium (0.14% yeast nitrogen base, 0.5% ammonium sulfate and 1% succinic acid; 2% (w/v) glucose; 0.077% Complete Supplement Mixture (CSM, ForMedium), pH set to 5.8 with NaOH or KOH) and incubated for 48 h at 30°C. For experiments where the removal of a specific media component was studied, the pre-culture was performed in absence of this component in order to deplete intracellular storages of the component in question. For experiments where alternative nitrogen sources were used, two consecutive pre-cultures were performed, the first containing low amounts of ammoniumsulfate (0.05%) in order to avoid excessive nitrogen storage, the second replacing ammoniumsulfate with the indicated nitrogen source in amounts corresponding to an equivalent, limiting number of nitrogen atoms. For experimental runs, precultures were diluted 35x to an OD of 0.03 - 0.15 in 350µL of SD medium and cultivated for 72h in a Bioscreen analyzer C (Growth curves Oy, Finland). Optical density was measured using a wide band (450-580 nm) filter. Incubation was at 30.0° C (± 0.1° C) with ten minutes preheating time. Plates were subjected to shaking at highest shaking intensity with 60s of shaking every other minute. OD measurements were taken every 20 minutes. Strains were run in duplicates on separate plates with ten BY4741 replicates in randomised (once) positions on each plate as reference. Plate layout was kept unchanged throughout the experimental series. Flocculation, which is a serious problem in liquid cultivations of wild yeast cells in higher cultivation volumes, was not observed.

**Data analysis:** Following visual inspection, severely flawed experiments, e.g. as due to lamp failure, were discarded and rerun. Optical density measurements (OD) were calibrated in the following way:

1. Background correction was achieved by subtracting the SD medium background of 0.067.
2. Non-linearity of optical density and population density at higher population densities was compensated for by calibration of each OD measurement as: calibrated OD = OD + 0.8324*OD^3^ .
3. Noise reduction was achieved by a moving average smoothening: each OD measurement, X_i,smoothened_ = (X_i-1_+X_i_+X_i+1_)/3.
4. Confounding effects from stationary phase proliferation curve collapses was compensated for by removing all negative slopes (if X_i+1_< X_i_ then set X_i+1_ = X_i_).

From calibrated proliferation curves, each fitness variable was calculated as:

Proliferation lag: Growth measurements were LN transformed, slopes were calculated between every pair of measurements spaced 140min apart, intercepts between each calculated slope and the extended horizontal line given by the start OD (average of initial five measurements) were calculated and the mean of the two highest intercepts was taken as the length of the lag phase. Proliferation lags longer than 48h were set to 48h.

Proliferation rate: Growth measurements were LN transformed. Slopes were calculated between every pair of measurements spaced 40min apart along the curve (no slopes were calculated from the eight initial time points to filter for digitalisation effects), the top two slopes were discarded to exclude possible artefacts and a mean was calculated from the third to the eight highest slopes. Population doubling time was calculated as LN(2) divided by the mean. Population doubling times longer than 48h were set to 48h.

Proliferation efficiency: The difference between end OD (last measurement) and initial OD (average of initial five measurements) was calculated and taken at face value as a measure of total change in population density. No proliferation efficiency was calculated from growth curves for which no stationary phase was reached, defined as curves with a coefficient of variation over the last five measurements >2%. Proliferation efficiency lower than 0.05 OD units were set to 0.05 OD units.

The calculated fitness variables were transferred onto log scale (Log_2_). To normalize fitness variables across plates, runs and instruments, each fitness variable was related to the corresponding measure from ten reference strains (BY4741) included on the same plate. If the BY4741 could not be used, due to defects associated with its auxotrophic markers, the progenitor strain S288C was used for normalization instead. Relative fitness measures for each strain and trait, LSC_ij_, were calculated as:

where wt_kj_ is the fitness variable of the k:th measurement of the wildtype for trait j, x_ij_ is the measure of strain i for trait j and r indicates the run. The LSC for proliferation efficiency was inverted in order to maintain directionality between fitness variables. *S. cerevisiae* strains RM-11, YJM789, YIIc17_E5 and W303 contained known or suspected auxotrophic markers and no measurements of proliferation could be made on these strains when cultivated without external additions of the relevant supplements.

**Associating phenotypes to CNVs, stop codons and novel genes:** For all *S. cerevisiae* isolates, copy number variations, stop codons and novel genes were extracted using previously published genomic sequence information.

Copy Number Variations: We reasoned that a Copy Number Variation for a gene would appear as an excess or deficit of reads from a particular strain corresponding to that gene. We therefore used BLAST to count the number of reads that matched each gene. Under the assumption that reads are uniform across the genome, the number of reads overlapping any particular point is expected to be Poisson distributed with a mean equal to the genome coverage.  The expected number of reads overlapping each gene is therefore simply proportional to the length of the gene (*L_g_*).  Because the sequencing coverage varied for each strain, we estimated a total coverage parameter (α) for the entire set of strains by regressing the total number of number of sequence reads matching (90% id for at least 300 bp, or 100bp if overhanging) each gene on the length of each gene, and then scaled it by the fraction of reads that came from a particular strain (*f_s_*). The expected number of reads (*n*) for single copy gene (*g*) in strain (*s*) is then:


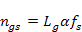


To estimate the average copy number (*c_g_*) for each gene, we compared the number of expected reads assuming that gene was single copy in all strains to the observed number of reads overlapping that gene (*x*).


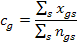


Therefore, the number of reads expected for each gene in each strain is *c_gs_n_gs_*. To identify genes whose number of reads (*x_gs_*) differed significantly from this, we computed the two-tailed *P*-value of observing a value more extreme than *x_gs_* from a Poisson distribution with parameter λ*_gs_* = *c_gs_* *n_gs_*. The *P*-values for each strain were then multiplied together, and 218 genes with product of *P*-values less than e^-50^ were considered copy number variable. Because under the null hypothesis the product of *k* *P*-values is known to follow a *χ*^2^ distribution with d.f.=2*k*, e^-50^ corresponds to *P*=0.047 for a product of 39 *P*-values. To test these genes for association with phenotypes, we computed a ‘copy number ratio’, *r_gs_*,


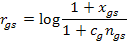


After excluding dubious genes and transposable elements (which represented the most highly variable genes), Spearman rank correlations were computed between the copy number ratios for 88 genes and 600 phenotypes. Because the phenotypes are highly correlated, and the number of individuals was too small to have statistical power, we explored this data using cluster analysis, rather than simply choosing a multiple testing correction threshold. We considered genes showing consistent correlations among different concentrations of the same toxin, or consistent changes in all experiments in a particular nutrient source, more likely to be reliable.

Stop codons**:** Stop codon mutations with a minor allele frequency of ≥3 were found in 63 genes, excluding dubious ORFs. Association between each gene and each phenotype was tested using the two tailed Students t-test as well as the two sample Kolmogorov-Smirnov test, both Bonferroni corrected. Those candidate associations that were significant in both tests at p-values p<0.015 in the t-test and p<0.15 in the K-S test were retained.

Novel genes: 44 genes not annotated in the S288c reference genome but present in at least two of the other isolates were identified as previously described . For each novel gene and each phenotype, significant association was tested using a Bonferroni corrected two tailed Students t-test; candidate associations significant at p<0.1 were retained.

Candidate associations were compared to a systematic linkage mapping of crosses between the clean lineages and were retained only if a significant QTL where detected in the corresponding chromosomal region in at least one of the crosses.

**SUPPORTING REFERENCES**

1. Liti G, Carter DM, Moses AM, Warringer J, Parts L, et al. (2009) Population genomics of domestic and wild yeasts. Nature 458: 337-341.

2. Brachmann CB, Davies A, Cost GJ, Caputo E, Li J, et al. (1998) Designer deletion strains derived from Saccharomyces cerevisiae S288C: a useful set of strains and plasmids for PCR-mediated gene disruption and other applications. Yeast 14: 115-132.

3. Cubillos FA, Louis EJ, Liti G (2009) Generation of a large set of genetically tractable haploid and diploid Saccharomyces strains. FEMS Yeast Res 9: 1217-1225.

4. Gietz RD, Woods RA (2002) Transformation of yeast by lithium acetate/single-stranded carrier DNA/polyethylene glycol method. Methods Enzymol 350: 87-96.

5. Warringer J, Anevski D, Liu B, Blomberg A (2008) Chemogenetic fingerprinting by analysis of cellular growth dynamics. BMC Chem Biol 8: 3.

6. Warringer J, Blomberg A (2003) Automated screening in environmental arrays allows analysis of quantitative phenotypic profiles in Saccharomyces cerevisiae. Yeast 20: 53-67.

7. Warringer J, Ericson E, Fernandez L, Nerman O, Blomberg A (2003) High-resolution yeast phenomics resolves different physiological features in the saline response. Proc Natl Acad Sci U S A 100: 15724-15729.

8. Cubillos FA, Billi E, Zorgo E, Parts L, Fargier P, et al. (2011) Assessing the complex architecture of polygenic traits in diverged yeast populations. Mol Ecol 20: 1401-1413.
